# Study on chloride ion erosion resistance of recycled aggregate concrete based on an improved TOPSIS model integrating entropy weight and AHP

**DOI:** 10.1371/journal.pone.0352439

**Published:** 2026-07-22

**Authors:** Yali Cao, Wenbang Zhu, Xinjie Wang, Chuikan Li, Ruiming Liu, Dali Zhang, Xiumei Zheng, Baochen Tang

**Affiliations:** 1 College of Civil Engineering, Kashi University, Kashi, China; 2 Xinjiang Key Laboratory of Engineering Materials and Structural Safety, Kashi University, Kashi, China; 3 Kashi Zhengxin Construction Engineering Testing Co., Ltd, Kashi, China; King Mongkut's University of Technology North Bangkok, THAILAND

## Abstract

Recycled coarse aggregate (RA) is prone to deteriorating the performance of recycled aggregate concrete (RAC) due to inherent defects such as adhered old cement paste and internal micro – cracks, while calcined layered double hydroxides (CLDHs) exhibit significant potential for enhancing concrete performance. However, the synergistic mechanism between CLDHs and RA remains unclear. To address this, this study employs compressive strength, chloride ion (Cl^-^) penetrability, X - ray diffraction (XRD), scanning electron microscopy (SEM), and nuclear magnetic resonance (NMR) to investigate the effects of varying CLDHs content (0%, 1%, 3%, 6%) and RA replacement rates (0%, 10%, 20%, 30%) on the mechanical properties, chloride ion permeability resistance, and microstructure of RAC. Results indicate that an appropriate combination of CLDHs and RA significantly improves RAC performance: the mix with 1% CLDHs and 20% RA increased the 28 d compressive strength by 10.3% compared to the reference group, while the combination of 3% CLDHs and 30% RA enhanced chloride ion penetration resistance by 19.1%, with electrical flux as low as approximately 1135 C, achieving a “low” permeability rating. Microstructural analysis confirms that the synergistic interaction of suitable CLDHs and RA promotes the formation of dense flocculent C – S – H gel, fills pores, reduces the proportion of harmful pores, increases the ratio of gel pores, thereby optimizing pore structure and enhancing system compactness. Additionally, it delays crack initiation and propagation, resulting in no penetrating cracks upon specimen failure. Based on these findings, an improved TOPSIS comprehensive evaluation model integrating the entropy weight (EW) method and the Analytic Hierarchy Process (AHP) was developed. This model systematically evaluates the chloride ion erosion resistance of Recycled Aggregate Concrete (RAC) by synthesizing multidimensional indicators such as compressive strength, electrical flux, and pore structure, thereby overcoming the limitation of single-factor weighting inherent in the entropy weight method. Concurrently, through economic analysis, an adjustable decision-making framework for RAC mix proportion selection under various corrosive environments has been proposed. This study elucidates the mechanism by which CLDHs and RA synergistically improve RAC performance, providing a theoretical foundation and methodological support for the engineering application of recycled concrete in aggressive environments.

## 1. Introduction

In the context of global urban intensification, the demand for natural sand and gravel aggregates continues to rise, resulting in excessive exploitation of natural resources and mounting pressure on ecological environments [1]. Concurrently, the demolition and renovation of buildings generate substantial amounts of waste concrete aggregates, whose accumulation increasingly poses significant challenges. Against this backdrop, the conversion of such aggregates into RAC has emerged as a pivotal strategy to alleviate the dual pressures of construction waste disposal and resource scarcity [2–3].

The development and utilization of RAC has garnered widespread attention. Research indicates that incorporating pozzolanic materials [4], mineral admixtures [5–7], and fibers [8–14] can, to some extent, enhance its mechanical properties and chloride penetration resistance. Nevertheless, RAC continues to exhibit certain inherent shortcomings, including high porosity and relatively weak resistance to chemical attack. CLDHs has drawn increasing interest due to its dual functionality of pore refinement and chloride ion binding via chemical reaction, and its synergistic interaction with RA holds potential for overcoming the limitations of conventional modification methods. Studies by Yang et al. [15–16] have confirmed that CLDHs exhibits good compatibility and stability within cementitious systems, effectively reducing mortar porosity and improving mechanical performance as well as chloride binding capacity. However, the synergistic mechanism between CLDHs and RA in concrete, along with the optimal mix proportion for maximizing impermeability, remains inadequately understood.

A series of studies have been conducted to investigate the influence of RA on the performance of concrete, with a particular focus on its substitution ratio. Pereira-de-Oliveira et al. [17] were among the first to validate the feasibility of reusing RA in concrete and noted that its substitution for natural aggregate affects the macroscopic properties of concrete. Subsequent research by Le et al. [18–20] indicated that, under a fixed water – cement ratio, compressive strength decreases as the substitution ratio of RA increases. Another study [21] revealed that the compressive strength of concrete reaches its optimum when the substitution ratio of RA is 30%, with the performance variation primarily attributable to adhered old mortar and pore structure. In terms of durability, research by Du et al. [22] demonstrated that the chloride ion diffusion coefficient increases linearly with a higher substitution ratio of RA. Meanwhile, Kwan et al. [23–24] pointed out that the permeability of concrete is closely related to its pore structure and microcracks; the porous nature of RA tends to exacerbate permeability, although this effect is negligible at low substitution ratios (<30%). Overall, existing studies provide insufficient mechanistic explanation of how RA influences concrete properties, and the mix design for RAC under multi – factor synergistic effects remains unclear.

The potential of CLDHs as a functional admixture in enhancing the performance of cement – based materials has been well documented. Research indicates that hydrotalcite nanoparticles can effectively regulate the cement hydration process and refine the pore structure, thereby significantly improving the mechanical strength and impermeability of the matrix [25–26]. For instance, Cao et al. [27] demonstrated through mercury intrusion porosimetry and mechanical testing that incorporating 2% calcined hydrotalcite increases compressive strength by 8.7%. Similarly, Long et al. [28–29] reported superior resistance to chloride ion penetration in concrete at this dosage. Microstructural investigations by He et al. [30] further revealed that this performance optimum stems from the synergistic effects of physical filling and chemical adsorption. However, existing studies have primarily focused on conventional concrete systems, leaving the optimal proportioning of CLDHs and its synergistic mechanism in RAC – a porous and heterogeneous system – insufficiently explored.

In the domain of concrete mix optimization and performance evaluation, the Technique for Order Preference by Similarity to Ideal Solution (TOPSIS) serves as a widely adopted methodology. Zhu et al. [31], while investigating ultra-high performance engineered cementitious composites incorporating waste rubber scraps, employed the TOPSIS method to evaluate their dynamic responses and overall performance under varying temperatures. Yang et al. [32] integrated TOPSIS with the AHP to achieve multi-objective optimization of concrete mix proportions for diaphragm walls and provided recommended formulations. Furthermore, Lu et al. [33] applied an EW – TOPSIS approach to identify the optimal mixture design when exploring the synergistic effects of fibers and expansion agents. In general, existing optimization processes predominantly rely on either singular objective data or subjective experiential judgments, with a notable scarcity of comprehensive evaluation models that integrate both subjective and objective weighting mechanisms.

In response to the aforementioned issues, this study formulates mix proportions with varying contents of calcined hydrotalcite and replacement ratios of recycled aggregates, and conducts mechanical performance and electric flux tests. Through microstructural analysis techniques such as XRD, SEM, and NMR, the mechanisms of strength enhancement and chloride resistance under synergistic effects are preliminarily elucidated. Furthermore, an improved TOPSIS model integrating entropy weight and AHP, along with an economic analysis model, are proposed to achieve a multi – criteria comprehensive evaluation and optimization of chloride ion erosion resistance. These findings provide theoretical foundations and technical support for producing high – performance recycled concrete in saline soil regions.

## 2. Materials and methods

### 2.1. Raw materials

**Fig 1:**
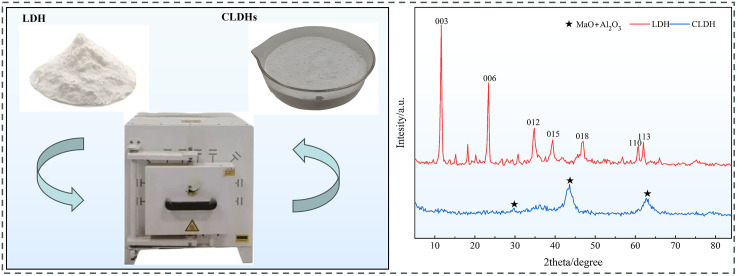
XRD diffraction patterns of hydrotalcite before and after calcination.

**Fig 2:**
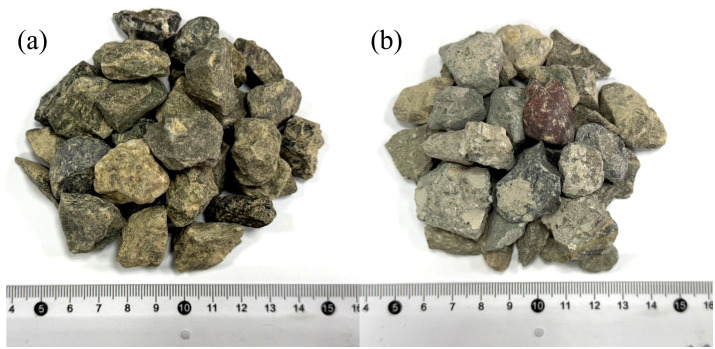
Coarse aggregate. (a) Crushed stone, (b) Recycled aggregate.

**Fig 3:**
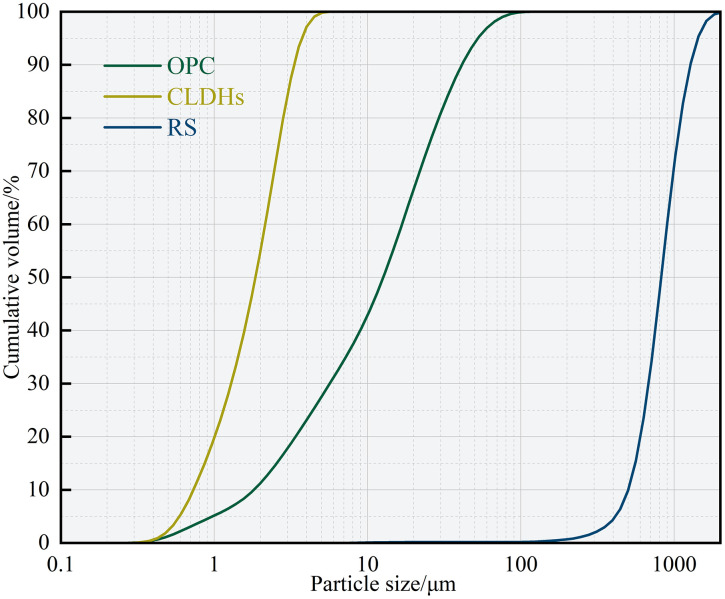
Laser particle size analysis results of CLDHs, OPC, and RS.

The 42.5 – grade ordinary Portland cement (OPC) employed was procured from Kashi Tianshan Cement Company, conforming to the GB/T 175–2023 standard [34].The hydrotalcite, of the magnesium – aluminum type, was supplied by Shandong Wanxin Weina Materials Technology Co., Ltd. The hydrotalcite was calcined in a muffle furnace using a heating rate of 5 °C/min, maintained at 500 °C for 5 h, and then cooled naturally to room temperature, yielding calcined layered double hydroxides (CLDHs) with the chemical formula Mg_6_Al_2_O_9_. Fig 1 presents the XRD patterns of hydrotalcite before and after calcination. After high – temperature treatment, the characteristic layered diffraction peaks (003 and 006) of hydrotalcite completely disappeared, and only weak diffraction signals corresponding to Mg – Al composite oxides were observed in the XRD pattern. The water – reducing agent, a standard – type agent produced by Sichuan Dongrun Baisheng New Materials Co., Ltd., exhibited a water reduction rate exceeding 25%. The chemical compositions of OPC and CLDHs were determined by X - ray fluorescence (XRF) spectroscopy, with results listed in Table 1. Mixing water was obtained from the local tap water supply in Kashi. Fine aggregate consisted of river sand (RS) sourced from Shufu County, Kashi. Coarse aggregate was composed of crushed stone and recycled aggregate blended at a specific ratio. The recycled aggregate (RA), derived from a demolished building, was processed via jaw crusher fragmentation and screening. Post – processing, both types of aggregate showed similar particle sizes, as illustrated in Fig 2. Physical properties of the crushed stone and recycled aggregate are provided in Table 2. Fig 3 presents the laser particle size distributions of CLDHs, OPC, and RS. Compared to OPC, CLDHs exhibit a higher specific surface area, with all three materials forming a well – defined particle size gradient distribution.

**Table 1:** Main chemical components of OPC and CLDHs (wt%).

Materials	SiO_2_	Al_2_O_3_	CaO	Fe_2_O_3_	MgO	K_2_O	SO_3_
OPC	25.4	7.1	55.6	4.9	1.3	1.4	3.3
CLDHs	1.1	40.9	2.7	0.1	53.8	0.01	1.0

**Table 2:** Coarse aggregate physical properties.

Coarse aggregate	Continuous gradation/ mm	Dry density/ g·cm^-3^	Water absorption/ %
Crushed stone	5 - 25	2.65	0.45
Recycled aggregate	5 - 25	2.26	3.2

### 2.2. Mix design

**Fig 4:**
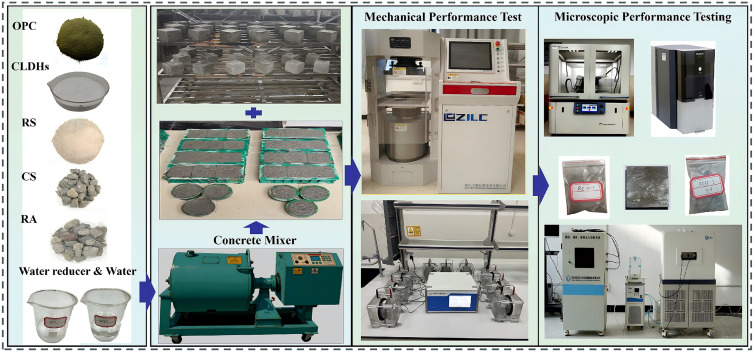
Preparation and testing process of RAC.

The mix design of Recycled Aggregate Concrete (RAC) was conducted in accordance with Chinese Standard JGJ 55–2011 [35], incorporating CLDHs dosage (0%, 1%, 3%, 6%) and RA replacement ratios (0%, 10%, 20%, 30%). Ten mix proportions are detailed in Table 3, wherein RA replacement ratio is defined by its proportion to coarse aggregate and CLDHs dosage by its proportion to the total binder materials. All raw materials were batched according to the predetermined ratios; aggregates and cementitious materials were first dry – mixed for 30 s, followed by the addition of pre – dissolved superplasticizer in water and wet mixing for an additional 2 min. After mixing, the fresh concrete was cast into molds to fabricate cylindrical specimens (Φ100 mm × 50 mm) and cubic specimens (100 mm × 100 mm × 100 mm), which were subsequently compacted using a vibrating table. The specimens were demolded after 24 h of curing under a plastic film at room temperature, then transferred to a standard curing environment maintained at 20 ± 2°C and relative humidity ≥95% for 28 d. The detailed preparation procedure is illustrated in Fig 4.

**Table 3:** Mix proportion design of RAC.

Specimen NO.	CLDHs/%	Recycled aggregate/%	Water-binder ratio	water reducer/%	Consumption of test materials/kg·m^-3^						
					OPC	CLDHs	Water	River sand	Crushed stone	Recycled aggregate	water reduce
RC0−0	0	0	0.4	1.5	420	0	168	580	1320	0	6.3
RC10−1	1	10	0.4	1.5	415.8	4.2	168	580	1188	132	6.3
RC10−3	3	10	0.4	1.5	407.4	12.6	168	580	1188	132	6.3
RC10−6	6	10	0.4	1.5	394.8	25.2	168	580	1188	132	6.3
RC20−1	1	20	0.4	1.5	415.8	4.2	168	580	1056	264	6.3
RC20−3	3	20	0.4	1.5	407.4	12.6	168	580	1056	264	6.3
RC20−6	6	20	0.4	1.5	394.8	25.2	168	580	1056	264	6.3
RC30−1	1	30	0.4	1.5	415.8	4.2	168	580	924	396	6.3
RC30−3	3	30	0.4	1.5	407.4	12.6	168	580	924	396	6.3
RC30−6	6	30	0.4	1.5	394.8	25.2	168	580	924	396	6.3

### 2.3. Test method

This study conducted compressive strength tests, chloride ion permeability tests, X-ray diffraction (XRD) analyses, scanning electron microscopy (SEM) observations, and nuclear magnetic resonance (NMR) measurements. The actual sample sizes for each experimental procedure are presented in Table 4.

**Table 4:** Actual sample sizes at each experimental phase.

Test item	Specimen specification/mm	Number of specimens per mix proportion group/pcs.	times of repetition/times
Compressive strength test (3d, 7d, 28d)	100 × 100	3	3
Chloride ion (Cl^-^) penetration test (28d)	Φ100 × 50	3	3
XRD test	Fine powder form	1	1
SEM test	Block	2	2
NMR test	40 × 40	3	3

#### 2.3.1. Compressive strength test.

The mechanical properties of RAC were evaluated in accordance with the Chinese standard GB/T 50081–2019 [36]. Cubic specimens measuring 100 mm × 100 mm × 100 mm were used for compressive strength tests, which were conducted at curing ages of 3 d, 7 d, and 28 d. The compressive strength of the specimens was determined under a constant loading rate of 0.5 MPa/s using a compression testing machine. The average value of the compressive strength results of one group of three cubes is used as the compressive strength test results. The strength of a single test block exceeds 10% of the average value and is removed.

#### 2.3.2. Chloride ion (Cl^-^) penetration test.

**Fig 5:**
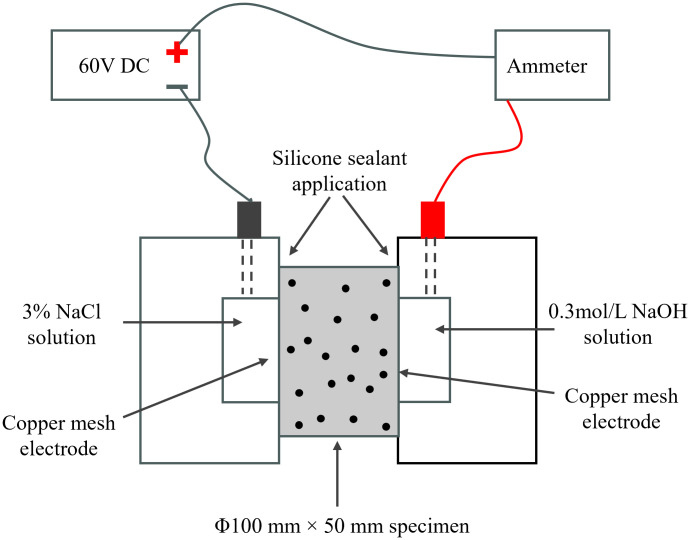
ASTM C1202 Standard test for electrical chloride penetration resistance.

The test was conducted in accordance with the Chinese standard GB/T 50082–2024 [37], using RAC cylindrical specimens with dimensions of Φ100 mm × 50 mm that were cured for 28 d. Firstly, the surface dust of the specimens was wiped off with a damp cloth and then left to air dry at room temperature until no free water was visible. Subsequently, the cylindrical sides of the specimens were sealed with epoxy resin to eliminate the interference of side penetration on the test results. After sealing, vacuum saturation treatment was carried out. The vacuum saturation operation strictly followed the following procedure: the sealed specimens were placed in a vacuum saturation device, the vacuum pump was started and the vacuum degree was adjusted to ≤133 Pa, and this vacuum state was maintained for 3 h. Keeping the vacuum condition unchanged, deionized water was slowly injected through the liquid injection port until the liquid level completely submerged all specimens. The specimens were then continuously soaked under normal pressure for 18 ± 2 h. After soaking, the specimens were taken out and the surface adhering water was gently wiped off with a damp cloth, and then temporarily stored in an environment with a relative humidity of ≥95%. To avoid the loss of water from the specimens affecting the test accuracy, the specimens should be placed in the chloride ion permeability test cell within 30 min. The DTL - 9T type concrete chloride ion permeability tester was used to conduct the test on the chloride ion penetration resistance performance. The specific test setup and operation process are shown in Fig 5.

#### 2.3.3. XRD test.

The phase composition of hydration products in RAC was analyzed using a TD – 3500 X - ray diffractometer(XRD). The sample preparation procedure was as follows: Samples were extracted from the RAC test block at a depth of 20 mm from the surface, subjected to vacuum drying, thoroughly ground in an agate mortar, and sieved through an 80 μm mesh to ensure uniform particle size distribution. The resulting powder was evenly spread and compacted in the sample holder to achieve a flat and dense testing surface. The XRD measurement parameters were configured as follows: Cu target X – ray source operated at 30 kV and 20 mA, with a scanning range from 5° to 85° at a rate of 0.1°/s. Continuous scanning mode was employed throughout the analysis to obtain well – defined characteristic diffraction peaks of the crystalline phases.

#### 2.3.4. SEM test.

The internal microstructure of RAC was examined and analyzed using a Phenom Pro X scanning electron microscope(SEM). The specimen preparation procedure was conducted as follows: Samples were extracted from the RAC test blocks at a depth of 20 mm from the surface and cut into rectangular specimens measuring 5 mm × 5 mm × 2 mm using a precision diamond saw. These specimens were then immersed in absolute ethanol for 24 h to terminate the hydration process, followed by vacuum drying. To mitigate charging effects induced by electron beam bombardment, a 5 ~ 10 nm thick gold coating was deposited on the specimen surface via sputter coating to enhance electrical conductivity.

#### 2.3.5 NMR test.

The internal pore structure parameters of RAC specimens with different mix proportions – including porosity, transverse relaxation time (T_2_) spectral distribution, and pore size distribution characteristics – were determined using a Phenom pro X scanning electron microscope in conjunction with a MesoMR12 - 060H - I high – precision nuclear magnetic resonance (NMR) concrete microstructure analyzer. The instrument provides an effective pore size measurement range of 0.00002 to 200 μm. The sample preparation and pretreatment procedures were conducted as follows: Cube specimens measuring 40 mm × 40 mm × 40 mm were drilled and cut from the RAC test blocks at a depth of 20 mm from the surface. The specimens were then placed into a vacuum saturation apparatus, where a vacuum was applied and maintained for 8h to eliminate air entrapped within the pores. While maintaining the vacuum, deionized water was introduced into the apparatus until the specimens were fully submerged. The samples continued to soak under vacuum for an additional 24 h to ensure thorough water saturation. Upon reaching full saturation, the specimens were removed for subsequent NMR testing.

## 3. Test results and discussions

### 3.1. Compressive strength

#### 3.1.1. Effects of CLDHs and RA on compressive strength of concrete.

**Fig 6:**
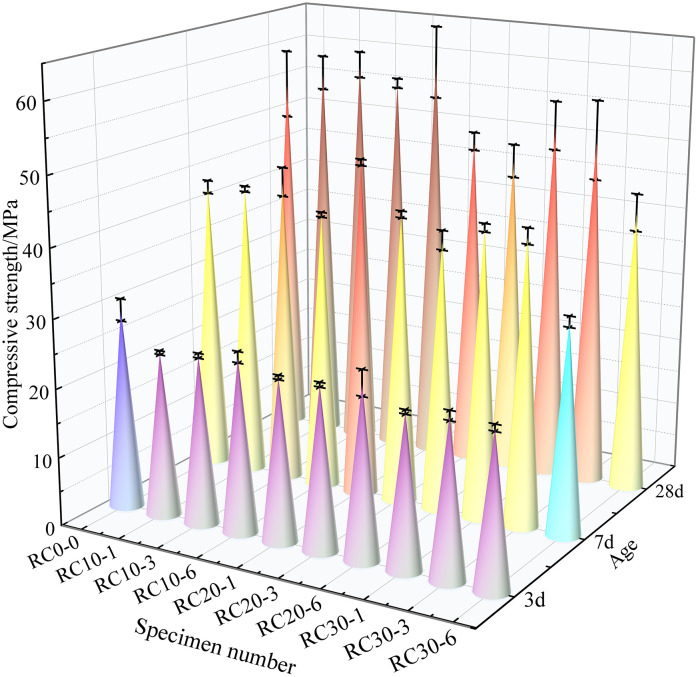
Relationship between compressive strength and CLDHs dosage.

Fig 6 illustrates the influence patterns of CLDHs dosage and RA replacement rate on the compressive properties of RAC specimens at different curing ages. At 3 d, the compressive strength of all RAC test groups was lower than that of the reference ordinary concrete. The primary reasons are as follows: CLDHs tend to absorb water during the early hydration stage, significantly inhibiting the hydration process of cementitious materials. Meanwhile, the interface transition zone between the old mortar adhered to the RA surface and the new cement exhibits inherent defects, compounded by the high water absorption of RA. The significance analysis revealed that both the replacement ratio of recycled aggregates and the dosage of CLDHs exerted a statistically significant influence on the compressive strength of recycled aggregate concrete at all curing ages. At the early age of 3 d, the compressive strength decreased significantly with increasing recycled aggregate content, with the reference group demonstrating markedly superior performance compared to all modified groups. As hydration progressed, the results at 7 and 28 d indicated that an appropriate combination of CLDHs and recycled aggregates could synergistically enhance the compressive strength of concrete [38]. However, excessive dosage of either component or an imbalanced ratio between the two led to a reduction in concrete strength. Among the tested mixtures, RC20−1 (1% CLDHs + 20% recycled aggregates) achieved the highest compressive strength, whereas RC30−6 (6% CLDHs + 30% recycled aggregates) yielded the lowest. At 7 d, the effect of CLDHs dosage became evident, with mixtures containing 1% CLDHs exhibiting superior strength performance. The RC20−1 group significantly outperformed other specimens, while groups with high levels of both recycled aggregates and CLDHs showed a pronounced decline in strength.

As illustrated in Fig 6, when the CLDHs dosage is 1%, the 7 d and 28 d compressive strength of RAC initially increases and then decreases with the rising RA replacement ratio. Compared with the control group RC0–0, the compressive strength of RC10–1 and RC20–1 increases by 1.2% and 15.6%, and 4.2% and 10.3%, respectively. This observation indicates that CLDHs particles effectively fill the microscopic pores within the concrete, improve the interfacial bonding performance between RA and cement paste, reduce porosity, and enhance concrete compactness. Moreover, the complex surface morphology of RA forms tight interlocking with fine aggregates, while the detached fine particles fill the interfacial transition zone, optimizing the continuity of the particle gradation. When the RA replacement ratio increases to 30%, the 28 d compressive strength of specimen RC30−1 decreases by 3.3% compared with the control group, which is primarily attributed to the increased proportion of RA at high replacement levels, leading to a significant rise in the number of interfacial transition zones between old and new mortar, thereby restricting the strength development of RAC.

**Fig 7:**
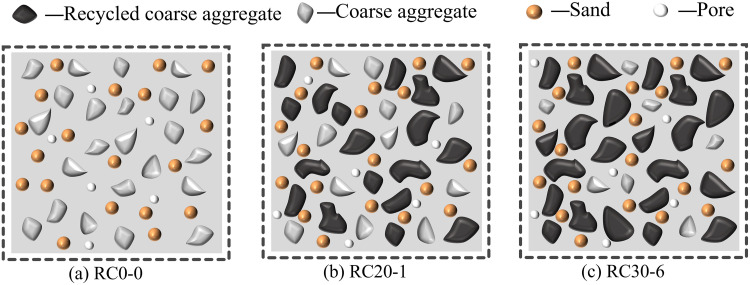
Mechanism of aggregate filling.

With the progressive increase in CLDHs dosage, the compressive strength of 7 d and 28 d RCA specimens exhibited varying degrees of reduction under the same RA replacement ratio. Compared to the control group, the strengths of RC20–3 and RC30–3 specimens decreased by 0% and 10.2% at 7 d, and by 1.2% and 5.9% at 28 d, respectively, indicating that CLDHs at this dosage can only partially improve the mechanical properties of RA. The long – term performance degradation of RA itself progressively becomes the dominant factor influencing compressive strength development. In contrast, specimens RC20–6 and RC30–6, with RA replacement ratios of 20% and 30%, exhibited significant strength deterioration, with reductions of 14.2% and 24.0%, respectively. These results demonstrate that a high dosage of CLDHs has a markedly adverse effect on the compressive performance of concrete. This can be attributed to the agglomeration tendency of excessive CLDHs, which reduces their dispersibility within the cementitious matrix. This phenomenon not only increases the proportion of large – diameter pores but also compromises the structural compactness of the concrete. Concurrently, the high CLDHs content leads to a relative reduction in OPC usage, resulting in insufficient formation of hydration products and ultimately causing a decline in strength [39]. At the 28 d core curing age, the groups with 10% recycled aggregate combined with 1% to 6% hydrotalcite, as well as those with 20% recycled aggregate and 1% hydrotalcite, exhibit no significant differences and demonstrate performance levels similar to that of the control group, representing the optimal performance. However, as the substitution rate of recycled aggregate increases to 30% and the hydrotalcite dosage rises to 3% to 6%, a notable decline in compressive strength is observed, with the RC30–6 group reaching the lowest value. This indicates that excessively high proportions of recycled aggregate and hydrotalcite exhibit a significant negative synergistic effect, thereby deteriorating the long-term mechanical properties of concrete. The associated aggregate mechanism is illustrated in Fig 7.

#### 3.1.2. Chloride ion(Cl^-^) penetration resistance of RAC.

A lower electrical flux corresponds to reduced chloride ion permeability, indicating superior resistance of concrete to chloride ingress. The ASTM C1202 rapid chloride permeability test method [40] is widely employed internationally for assessing concrete permeability, with the corresponding evaluation criteria outlined in Table 5.

**Table 5:** Chloride ion(Cl^-^) penetration assessment table for RAC.

Electric flux/C	Grade of concrete permeability
>4000	high
2000-4000	moderate
1000-2000	low
100-1000	very low
<100	negligible

**Fig 8:**
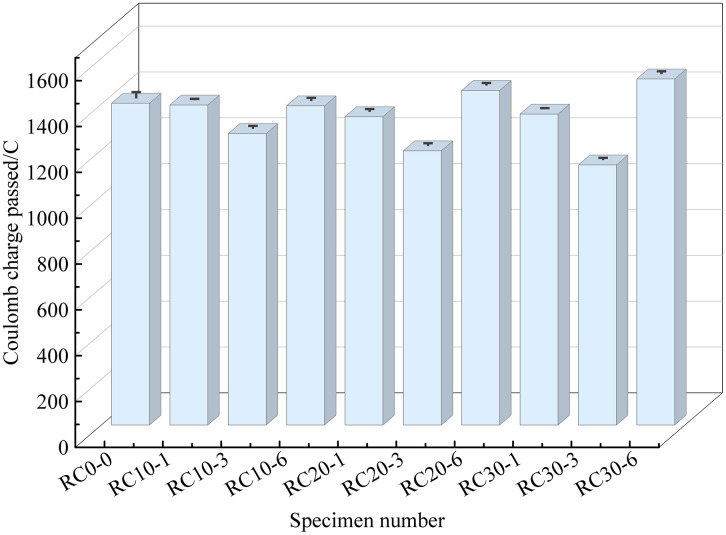
Relationship between electrical flux and CLDHs.

Fig 8 presents the electrical flux test results of RAC at 28 d with varying contents of CLDHs. As illustrated in Fig 8, the chloride ion penetration resistance of RAC initially increases and subsequently decreases with increasing CLDHs content. Among the tested specimens, the mix RC30–3, with 30% RA replacement and 3% CLDHs content, exhibits the lowest electrical flux value of approximately 1135 C, indicating a 19.1% improvement in chloride ion penetration resistance and representing the optimal performance. In contrast, the RC30–6 mix, with 30% RA replacement and 6% CLDHs content, shows the highest electrical flux value of 1510.2 C, corresponding to a low permeability grade, which implies relatively poor resistance to chloride ion penetration.The significance analysis reveals that both the replacement ratio of recycled aggregate and the dosage of calcined hydrotalcite exert a statistically significant influence on the electric flux of RAC. Under the same recycled aggregate replacement ratio, the electric flux of the group with 3% hydrotalcite dosage is significantly lower than that of the groups with 1% and 6% dosages. Specifically, the RC30–3 group exhibits the lowest electric flux, indicating superior resistance to chloride ion penetration. Conversely, the electric flux of the 6% hydrotalcite dosage group is markedly higher than that of the control group, demonstrating a significant deterioration in impermeability performance.

At a low dosage of 1% CLDHs, the specimen with a 10% RA replacement rate exhibited inferior impermeability due to the initially high compactness of the matrix and inadequate pore filling by CLDHs. Conversely, specimens with a 20% RA replacement rate demonstrated a reduction in electric flux, indicating that an appropriate RA replacement level promotes the formation of fine particles from adhered old mortar on the RA surface after crushing, which synergistically contributes to pore – filling alongside CLDHs. For specimens with a 30% RA replacement rate, however, the predominance of adhered old mortar on the RA surface slightly diminished the modifying effect of CLDHs.

When the CLDHs dosage was increased to 3%, the electric flux decreased by 9.4%, 14.8%, and 19.2% for specimens with RA replacement rates of 10%, 20%, and 30%, respectively. This result suggests that, at this stage, the pore – refining effect and chloride ion adsorption capacity of CLDHs become predominant, significantly enhancing the impermeability of the concrete. At a 3% hydrotalcite dosage, the electric flux exhibited a significant decreasing trend with increasing recycled aggregate replacement ratio. The combined use of high replacement ratios and 3% hydrotalcite substantially enhanced the impermeability.

However, when the CLDHs content reached 6%, the improvement in impermeability across all specimen groups was markedly reduced compared to other experimental sets. This attenuation is attributed to the aggravated “agglomeration effect” of CLDHs at high concentrations, which introduces new structural defects, increases porosity, and induces microcracks, thereby compromising the integrity and continuity of the concrete pore structure and consequently diminishing the resistance to chloride ion migration. At a 6% hydrotalcite dosage, the electric flux increased significantly with rising replacement ratios, indicating a negative synergistic effect. Specimens with 10% recycled aggregate combined with either 1% or 6% hydrotalcite showed no significant difference in electric flux compared to the control group. In contrast, those with 20% or 30% recycled aggregate combined with 1% hydrotalcite exhibited significantly lower electric flux and superior impermeability relative to the control.

**Fig 9:**
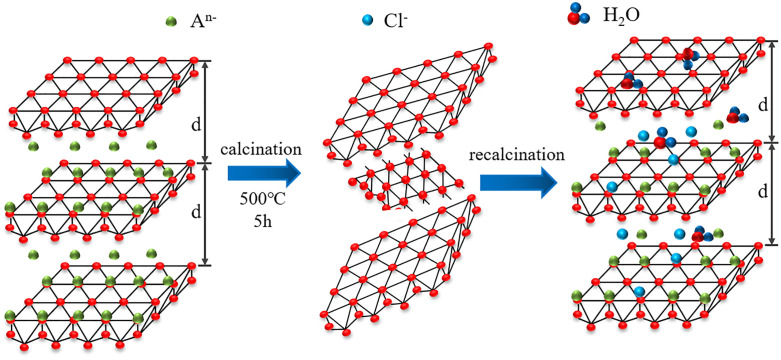
Mechanism of ion exchange in CLDHs.

The aforementioned results corroborate the advantages of CLDHs as a novel nano – modified material: they integrate both filler effect and lamellar memory effect, significantly enhancing concrete compactness and reducing chloride ion transport channels through dual modification mechanisms [41]. Concurrently, for chloride ions inherently present in RA, CLDHs effectively immobilize these ions at the source by leveraging their exceptional adsorption capacity, thereby mitigating chloride diffusion and corrosion behaviors, as illustrated in Fig 9. Furthermore, the synergistic interaction between CLDHs and RA remains a critical determinant of the differential electric flux observed across specimen groups. An appropriate dosage of CLDHs, when optimally aligned with the RA replacement ratio, can effectively diminish internal cracks and pores in concrete, resulting in a more densely structured matrix.

### 3.2. Micro-analysis

#### 3.2.1. XRD analysis.

To further investigate the influence mechanisms of RA and CLDHs on the mechanical properties and chloride ion penetration resistance of RAC, XRD analysis was employed to characterize the phase composition of RAC at 3 d and 28 d curing ages under varying CLDHs dosages and RA replacement ratios at the microstructural level.

**Fig 10:**
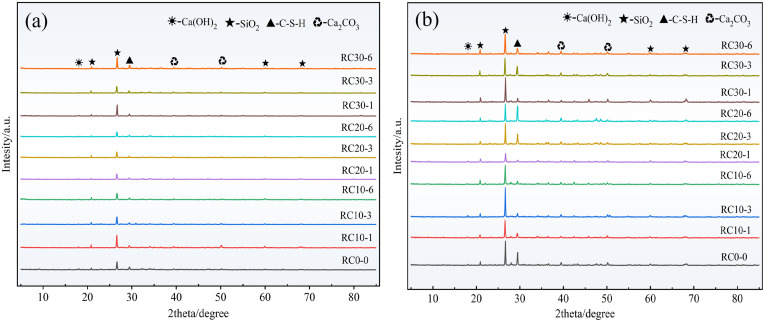
X –ray diffraction pattern of RAC. (a) RAC of 3 d, (b) RAC of 28 d.

As illustrated in Fig 10 display the XRD diffraction patterns of all experimental groups at curing ages of 3 d and 28 d, respectively. At the 3 d curing age, which corresponds to the early hydration stage, the intensities of diffraction peaks for different mix proportions show minor variations, indicating comparable degrees of early hydration across all groups. The predominant phases identified include Ca(OH)_2_, SiO_2_, and minor amounts of C – S – H gel.

Compared with the 3 d specimens, the hydration reactions at 28 d proceed more thoroughly, leading to substantial formation of C – S – H gel. Furthermore, Ca(OH)_2_ can activate the reactive SiO_2_ present in the old mortar attached to the RA, inducing a secondary hydration reaction. This observation reflects the synergistic effect between CLDHs and RA, which contributes to the sustained advancement of the hydration process.

In this context, the diffraction peak intensity of Ca(OH)_2_ for RC20–1 falls between that of RC0–0 and RC30–6, indicating that the hydration reaction of this group has reached a “golden equilibrium point”: a dosage of 1% CLDHs significantly promotes the formation of C-S-H gel through the nano – nucleation effect while maintaining the necessary conditions for adequate cement hydration, thereby forming a dense microstructure. In contrast, the 6% CLDHs dosage in RC30–6 is excessively high, leading to an insufficient effective water – cement ratio in the system, which suppresses the overall hydration process of cement. This results in an inadequate total amount of hydration products and ultimately compromises the compressive strength.

As illustrated in the figure, RC30–3 exhibits the weakest diffraction peak intensity of Ca(OH)_2_ and more prominent characteristic peaks of C – S – H gel, indicating that a 3% CLDHs dosage promotes the transformation of Ca(OH)_2_ into dense C – S – H gel through nanofilling and nucleation-inducing effects, thereby refining the pore structure and blocking the physical migration pathways of chloride ions. Moreover, CLDHs possess a high specific surface area and a unique layered crystal structure with positively charged surfaces, enabling the physical adsorption of free chloride ions via electrostatic interactions [29], which reduces the chloride ion concentration in the pore solution. Additionally, the interlayer anions of CLDHs can undergo ion exchange with chloride ions, leading to chemical immobilization of chloride ions within the crystal structure. The aforementioned phase evolution mechanism elucidates the underlying reasons for the differences in compressive strength and chloride ion penetration resistance of RAC under various combinations of CLDHs content and RA replacement ratios.

#### 3.2.2. SEM analysis.

**Fig 11:**
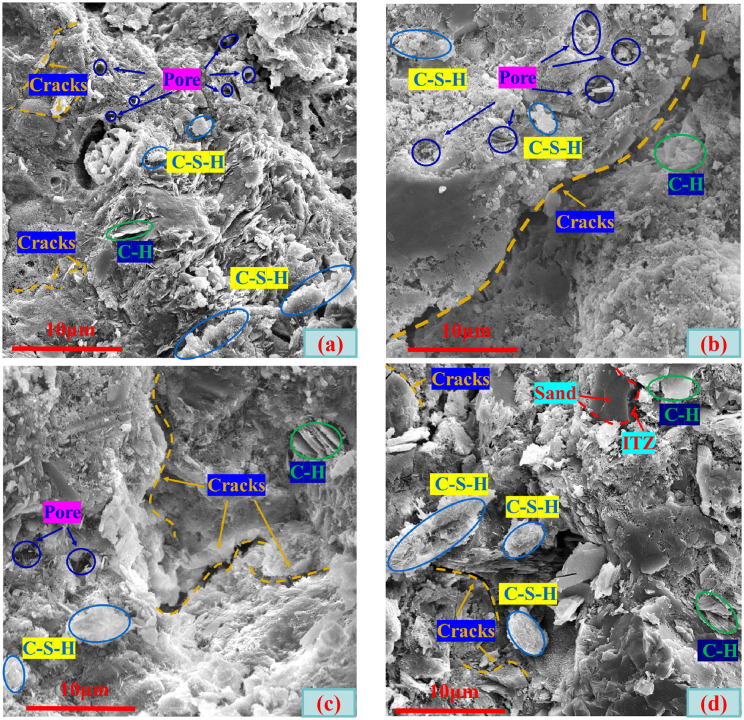
SEM micrographs of hydration products in RAC. (a) RC0−0, (b) RC20−1, (c) RC30−3, (d) RC30−6**.**

**Fig 12:**
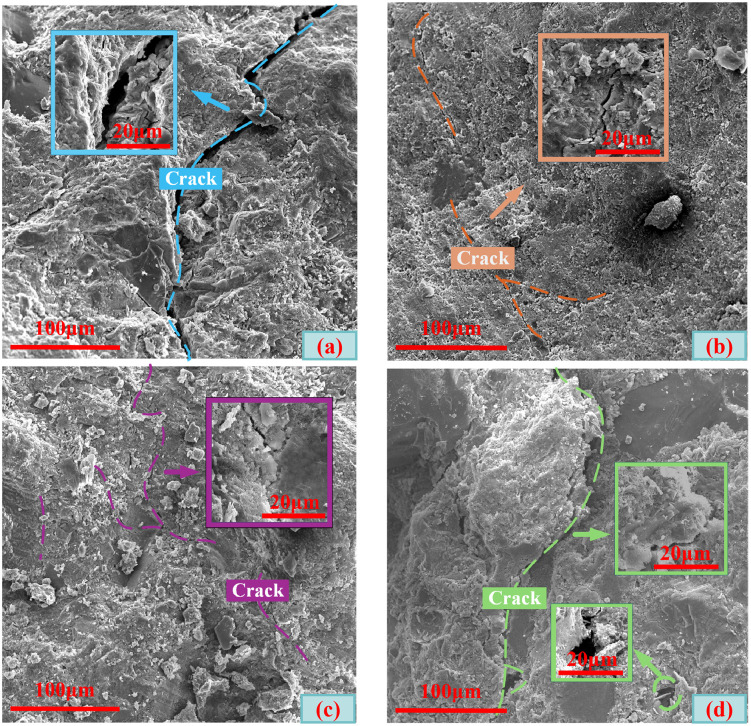
SEM Micrographs of cracks in 28 d of RAC. (a) RC0−0, (b) RC20−1, (c) RC30−3, (d) RC30−6.

To elucidate the intrinsic relationship between the macroscopic properties and microstructure of RAC, SEM was performed on representative specimens, as illustrated in Figs 11 and 12. The results indicate that the density of the microstructure serves as the fundamental determinant of compressive strength and chloride ion permeability resistance.

From the perspective of hydration product morphology, the dosage of CLDHs is a critical factor governing structural compactness. Fig 11 indicates that the reference group RC0–0, characterized by a high proportion of internal voids and microcracks, exhibits a loose structure, resulting in relatively low compressive strength. In contrast, the incorporation of 1% CLDHs (RC20−1) promotes the formation of abundant continuous and dense clusters of C – S – H gel, which significantly fills the pores, representing the key microstructural factor for its optimal macroscopic strength. However, when the CLDHs content increases to 6%, excessive addition leads to a pore – dominated and gel – dispersed microstructure, forming discontinuous frameworks that not only compromise mechanical strength but also provide pathways for chloride ion migration, resulting in a drastic performance decline. In comparison, the RC30–3 specimen with 3% CLDHs content develops dense flocculent gel phases that partially optimize the microstructure, thereby enhancing impermeability.

Analysis of the fracture development characteristics reveals that the morphology of cracks directly influences the permeability pathways of the material. A comparative examination of Fig 12 shows pronounced macroscopic cracks in RC0–0, characterized by considerable width and extensive propagation. Local magnification reveals rough crack surfaces with limited hydration products, indicating intense micro – cracking and inferior compressive performance. In contrast, RC20–1 specimens exhibit markedly reduced crack continuity and width compared to RC0–0, suggesting that the incorporation of CLDHs effectively restrains crack propagation. While the crack morphology of RC30–6 shows improvement over RC0–0, the presence of combined “pore - crack” defects significantly compromises matrix continuity, leading to lower compressive strength and chloride resistance than RC0–0. Overall, the crack and defect characteristics across RC0–0, RC30–3, and RC30–6 follow a pattern of “wide - narrow - relatively wide and porous”, which correlates directly with the resistance to chloride ion penetration. This microstructure observation explains the variation in chloride permeability resistance among the mixes: RC30–3 exhibits superior impermeability due to fine and discontinuous cracks, whereas RC0–0 and RC30–6, with wide cracks or composite defects, constitute vulnerable pathways for penetration. These microstructural features align well with the macroscopic chloride resistance performance.

#### 3.2.3. NMR analysis.

(1) T_2_ spectrum

**Fig 13:**
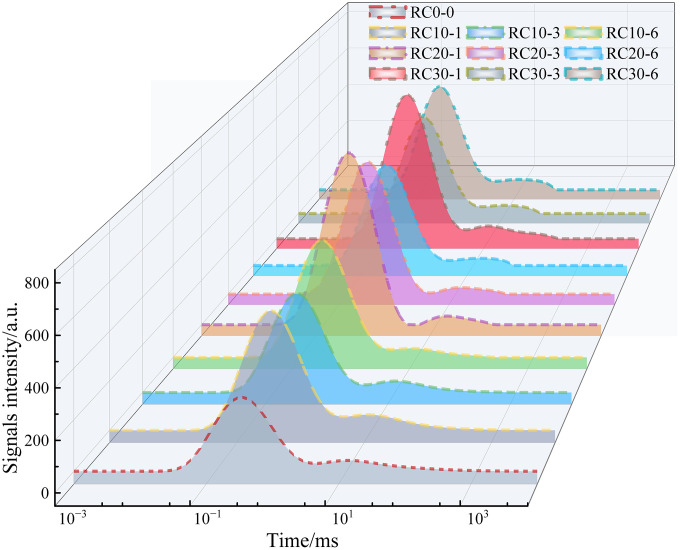
Temporal evolution profile of T_2_ relaxation time over 28 d.

The NMR T_2_ spectrum of RAC is illustrated in Fig 13. As observed in the spectrum, with increasing CLDHs content, the area of the T_2_ spectral peak in the relaxation time range of 0.7 - 2.3 ms (corresponding to small pores) generally exhibits a gradual decreasing trend. This suggests that when the CLDHs content is 1%, the specimens contain a relatively higher number of small pores, indicating that the CLDHs content significantly influences the pore characteristics of concrete [42].

Further comparison of the signal corresponding to large pores (within the relaxation time range of 300–1200 ms) reveals that only the control groups RC0−0 and RC30−6 (with 30% RA replacement rate and 6% CLDHs dosage) exhibit relatively high peak signal intensities, while those of the other groups decrease markedly. This demonstrates that the secondary hydration products of CLDHs effectively seal and segment cracks and inherent large pores introduced by RA. When the CLDHs content reaches 3%, this sealing and segmentation effect is sufficient to eliminate the majority of detectable large pores. However, the RC30–6 group still exhibits residual large – pore signals, implying that agglomeration occurs at a 6% CLDHs dosage, failing to sufficiently optimize the large – pore defects arising from the intrinsic porosity associated with 30% high replacement rate of RA.

(2) Pore size distribution

**Fig 14:**
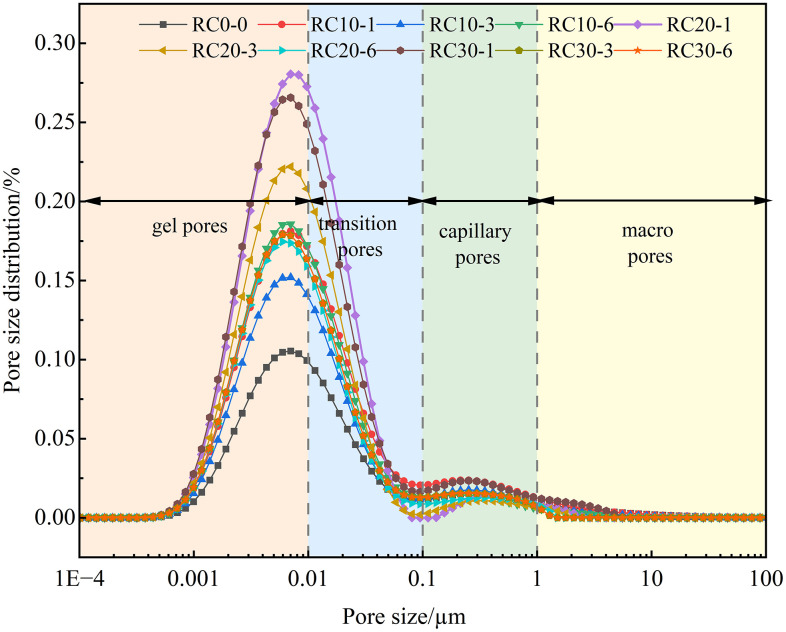
Aperture size distribution at 28 d.

To elucidate the underlying mechanisms governing T_2_ relaxation times, a refined analysis was conducted on the pore size distribution characteristics of RAC, as illustrated in Fig 14. The findings indicate that the pore size distribution is jointly modulated by the dosage of CLDHs and the replacement ratio of RA.

Maintaining a constant dosage of CLDHs, as the RA replacement ratio increases, the proportion of macropores in RC20–1 (20% RA replacement ratio, 1% CLDHs dosage) decreases by 16.5% compared with RC10–1 (10% RA replacement ratio, 1% CLDHs dosage), while the macropore proportion of RC30–1 (30% RA replacement ratio, 1% CLDHs dosage) further decreases by 32.6% relative to RC20–1. When the RA replacement ratio remains unchanged, the peak number of micropores in RC30–1 increases by 5.9% compared to the reference group, whereas RC30–6 (30% RA replacement ratio, 6% CLDHs dosage) shows a reduction of 4.7% compared to RC30−1. Additionally, RC30–3 (30% RA replacement ratio, 3% CLDHs dosage) exhibits a pore structure predominantly composed of finely dispersed small – sized pores with poor connectivity, which significantly impedes chloride ion diffusion. In contrast, RC30–6 possesses a higher porosity, with a more distributed and interconnected network in both capillary and macropore ranges, thereby facilitating pathways for chloride ion penetration.

In summary, the pore structure of concrete is jointly influenced by the synergy between the replacement ratio of RA and the dosage of CLDHs. When the CLDHs dosage remains constant, the number of large pores is minimized at a 30% RA replacement ratio. At a fixed RA replacement ratio, a 1% CLDHs dosage significantly increases the proportions of gel pores and transition pores, thereby refining the pore structure. However, an excessive dosage (e.g., 6%) leads to particle agglomeration, resulting in an increase in large pores and enhanced connectivity, which compromises the resistance to chloride ion penetration. The study by Yang et al. [43] on the influence of CLDHs on the pore structure and performance of concrete yielded results consistent with the present findings, further validating the reliability of the conclusions drawn in this work.

(3) Pore classification

To more precisely evaluate the influence of pores on the chloride ion erosion resistance of concrete, the pore hazard classification criteria from reference [44] were adopted: pores with radii less than 0.02 μm are classified as harmless pores, those between 0.02 μm and 0.05 μm as slightly harmful pores, those between 0.05 μm and 0.2 μm as harmful pores, and those greater than 0.2 μm as highly harmful pores.

**Fig 15:**
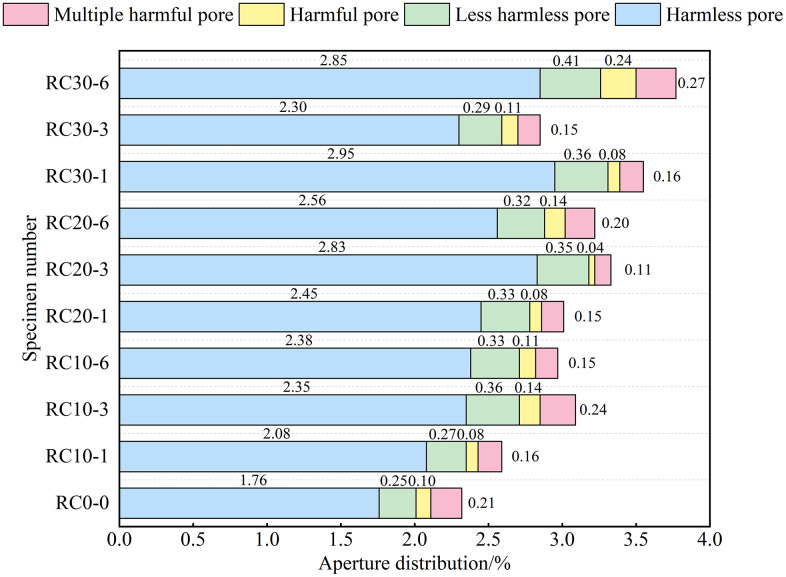
Aperture classification of RAC.

The statistical classification results of harmful pores in RCA specimens with different mix proportions are presented in Fig 15. As can be observed, compared to the reference group RC0–0, the proportion of highly harmful pores in concrete generally decreased after the incorporation of CLDHs, with the exception of RC30–6, which exhibited a relatively similar proportion. This result indicates that an appropriate dosage of CLDHs can significantly reduce the proportion of highly harmful pores and effectively optimize the pore structure characteristics. These findings are consistent with the results obtained from mechanical performance tests and electrical flux measurements of concrete.

## 4. Optimization scheme for chloride ion erosion resistance of RAC based on EW + AHP – TOPSIS method

### 4.1. EW – TOPSIS model

#### 4.1.1. Computational procedures of the EW – TOPSIS model.

The Entropy Weight (EW) objectively calculates the weight of each evaluation index based on the dispersion degree of measured data, thereby accurately reflecting the inherent discriminability of the data itself [45]. The Technique for Order Preference by Similarity to Ideal Solution (TOPSIS) method ranks alternatives by measuring their distances from the positive and negative ideal solutions, offering an intuitive evaluation of relative superiority [46]. The steps for applying the EW – TOPSIS model to optimize the selection of concrete resistance to chloride ion erosion are as follows.

(1) Determine the evaluation objects and indicators for the resistance to chloride ion penetration. Suppose there are m evaluation objects and n evaluation indicators for the resistance to chloride ion penetration of concrete, and the initial decision matrix *X* = *x*_*ij*_ is obtained, as shown in Eq. (1).

$$\begin{array}{c} X=[\begin{array}{cccc} x_{11} & x_{12} & \cdots & x_{1n}\\ x_{21} & x_{22} & \cdots & x_{2n}\\ \vdots & \vdots & \ddots & \vdots \\ x_{m1} & x_{m2} & \cdots & x_{mn} \end{array}] \end{array}$$ (1)

In the equation:*i*=1,2,…*m*;*j*=1,2,…*n*.

(2) The decision matrix *X* for the chloride ion erosion resistance of concrete is subjected to dimensionless processing to obtain the dimensionless matrix *Y* = *y*_*ij*_, wherein the evaluation indices for chloride ion erosion resistance are categorized into positive and negative indicators, as shown in Eqs. (2) to (3).

$$\begin{array}{c} Y_{ij}=[\begin{array}{cccc} y_{11} & y_{12} & \cdots & y_{1n}\\ y_{21} & y_{22} & \cdots & y_{2n}\\ \vdots & \vdots & \ddots & \vdots \\ y_{m1} & y_{m2} & \cdots & y_{mn} \end{array}] \end{array}$$ (2)

$$\begin{array}{c} \left\{ \begin{array}{c} @l@y_{ij}=\frac{x_{ij}-{\left(x_{ij}\right)}_{min}^{j}}{{\left(x_{ij}\right)}_{max}^{j}-{\left(x_{ij}\right)}_{min}^{j}},\left(\text{Positive indexes}\right)\\ y_{ij}=\frac{{\left(x_{ij}\right)}_{max}^{j}-x_{ij}}{{\left(x_{ij}\right)}_{max}^{j}-{\left(x_{ij}\right)}_{min}^{j}},\left(\text{Negative indexes}\right) \end{array}\right. \end{array}$$ (3)

In the equation: ${\left(x_{ij}\right)}_{min}^{j}$ represents the minimum value in the *j*-th column, while ${\left(x_{ij}\right)}_{max}^{j}$ denotes the maximum value in the *j*-th column.

(3) Calculate the weight matrix *P = p*_*ij*_ and the information entropy matrix *E* = *e*_*j*_ for each chloride ion erosion resistance performance indicator of concrete. A higher information entropy of a chloride ion erosion resistance indicator corresponds to a smaller amount of information carried and a lower utility value of information, as shown in Eqs. (4) to (5).

$$\begin{array}{c} e_{j}=-\frac{\text{1}}{lnm}\sum_{i=1}^{m}p_{ij}lnp_{ij} \end{array}$$ (4)

$$\begin{array}{c} p_{ij}=\frac{y_{ij}}{\sum_{i=1}^{m}y_{ij}} \end{array}$$ (5)

(4) Calculate the weight matrix *W*_*1*_ = *w*_*1j*_. The information utility value d_j_ of the chloride ion erosion resistance index increases with its importance, as shown in Eq. (6).

$$\begin{array}{c} w_{1j}=\frac{d_{j}}{\sum_{i=1}^{m}d_{j}} \end{array}$$ (6)

In the equation:The informational utility value of the *j*-th chloride ion erosion resistance index for concrete is defined as *d*_*j*_ = 1 – *e*_*j*_.

(5) Construct the decision matrix *V* = *v*_*ij*_ as specified in Eq. (7).

$$\begin{array}{c} v_{ij}=w_{1j}\cdot y_{ij} \end{array}$$ (7)

(6) Determining the positive and negative ideal solutions. The positive ideal solution (*V*^*+*^) represents the optimal value among all alternatives for each indicator, while the negative ideal solution (*V*^*-*^) corresponds to the worst value among all alternatives for each indicator, as expressed in Eqs. (8) to (9).

$$\begin{array}{c} V^{+}=\left\{v_{\text{1}}^{+},v_{\text{2}}^{+},\cdots ,v_{n}^{+}\right\} \end{array}$$ (8)

$$\begin{array}{c} V^{-}=\left\{v_{\text{1}}^{-},v_{\text{2}}^{-},\cdots ,v_{n}^{-}\right\} \end{array}$$ (9)

(7) Determine the Euclidean distance. The distance from each alternative's standardized and weighted vector *v*_*ij*_ to the positive ideal solution *V*^*+*^ is denoted as *S*^*+*^, while the distance to the negative ideal solution *V*^*-*^ is designated as *S*^*-*^, as illustrated in Eqs. (10) to (11).

$$\begin{array}{c} S^{+}=\sqrt{\sum_{j=1}^{n}{\left(v_{ij}-v_{j}^{+}\right)}^{\text{2}}} \end{array}$$ (10)

$$\begin{array}{c} S^{-}=\sqrt{\sum_{j=1}^{n}{\left(v_{ij}-v_{j}^{-}\right)}^{\text{2}}} \end{array}$$ (11)

(8) Compute the relative closeness *C*_*i*_, as illustrated in Eq. (12).

$$\begin{array}{c} C_{i}=\frac{S^{-}}{S^{+}+S^{-}} \end{array}$$ (12)

The optimal results were obtained, indicating that the ranking of chloride ion erosion resistance can be determined based on the magnitude of *C*_*i*_. A higher *C*_*i*_ value corresponds to superior resistance of concrete against chloride ion attack.

#### 4.1.2. Optimal selection of anti – chloride ion erosion performance schemes based on the EW – TOPSIS model.

In addressing the performance requirements of CLDHs - enhanced RAC in chloride environments, key indicators for evaluating chloride ion erosion resistance were selected: compressive strength, electrical flux, harmless pore, less harmless pore, harmful pore, and multiple harmful pore. A total of 10 candidate formulations of CLDHs - enhanced RAC were considered. An initial matrix was established and normalized to obtain the standardized matrix *Y*. Subsequently, entropy method analysis was applied to derive the dot product of the standardized matrix *Y* and the weight vector *W*_1_, forming the weighted normalized decision matrix *V*_*EW*_, thereby incorporating objective weights into the decision – making framework.


$$\begin{array}{c} Y=\left[\begin{array}{cccccc} @cccccc@0.70 & 0.28 & 1.00 & 1.00 & 0.70 & 0.38\\ 0.82 & 0.30 & 0.73 & 0.88 & 0.80 & 0.69\\ 0.92 & 0.64 & 0.50 & 0.31 & 0.50 & 0.19\\ 0.80 & 0.31 & 0.48 & 0.50 & 0.65 & 0.75\\ 1.00 & 0.44 & 0.42 & 0.50 & 0.80 & 0.75\\ 0.40 & 0.84 & 0.10 & 0.38 & 1.00 & 1.00\\ 0.28 & 0.13 & 0.33 & 0.56 & 0.50 & 0.44\\ 0.60 & 0.63 & 0.00 & 0.31 & 0.80 & 0.69\\ 0.53 & 1.00 & 0.55 & 0.75 & 0.65 & 0.75\\ 0.00 & 0.00 & 0.08 & 0.00 & 0.00 & 0.00 \end{array}\right] \end{array}$$

Based on matrix *Y*, the information entropy value *e*_*j*_ for each key indicator can be calculated, yielding matrix *E*.


$$\begin{array}{c} E=\left[\begin{array}{cccccc} @cccccc@0.93 & 0.81 & 0.87 & 0.92 & 0.94 & 0.92 \end{array}\right] \end{array}$$

Based on matrix *E*, the weight values of key indicators can be calculated and recorded as *W*_1_.


$$\begin{array}{c} W_{\text{1}}=\left[\begin{array}{cccccc} @cccccc@0.12 & 0.31 & 0.21 & 0.13 & 0.09 & 0.13 \end{array}\right] \end{array}$$

The aforementioned findings indicate that electrical flux constitutes the most critical influencing factor in the design of RAC. It is evident that electrical flux serves as a superior performance indicator over strength parameters for evaluating chloride ion penetration resistance.


$$\begin{array}{c} V_{EW}=\left[\begin{array}{cccccc} @cccccc@0.08 & 0.10 & 0.20 & 0.13 & 0.06 & 0.05\\ 0.09 & 0.18 & 0.15 & 0.11 & 0.07 & 0.09\\ 0.11 & 0.21 & 0.10 & 0.04 & 0.04 & 0.02\\ 0.09 & 0.11 & 0.10 & 0.06 & 0.06 & 0.10\\ 0.12 & 0.15 & 0.08 & 0.06 & 0.07 & 0.10\\ 0.05 & 0.28 & 0.02 & 0.05 & 0.09 & 0.13\\ 0.03 & 0.05 & 0.07 & 0.07 & 0.04 & 0.06\\ 0.07 & 0.14 & 0.00 & 0.04 & 0.07 & 0.09\\ 0.06 & 0.34 & 0.11 & 0.10 & 0.06 & 0.10\\ 0.00 & 0.00 & 0.02 & 0.00 & 0.00 & 0.00 \end{array}\right] \end{array}$$

Subsequently, the positive ideal solution (*V*_*EW*_^*+*^) and the negative ideal solution (*V*_*EW*_^*-*^) are identified from the matrix *V*_*EW*_.


$$\begin{array}{c} {V_{EW}}^{+}=\left[\begin{array}{cccccc} @cccccc@0.12 & 0.34 & 0.20 & 0.13 & 0.09 & 0.13 \end{array}\right] \end{array}$$

$$\begin{array}{c} {V_{EW}}^{-}=\left[\begin{array}{cccccc} @cccccc@0.00 & 0.00 & 0.00 & 0.00 & 0.00 & 0.00 \end{array}\right] \end{array}$$

**Fig 16:**
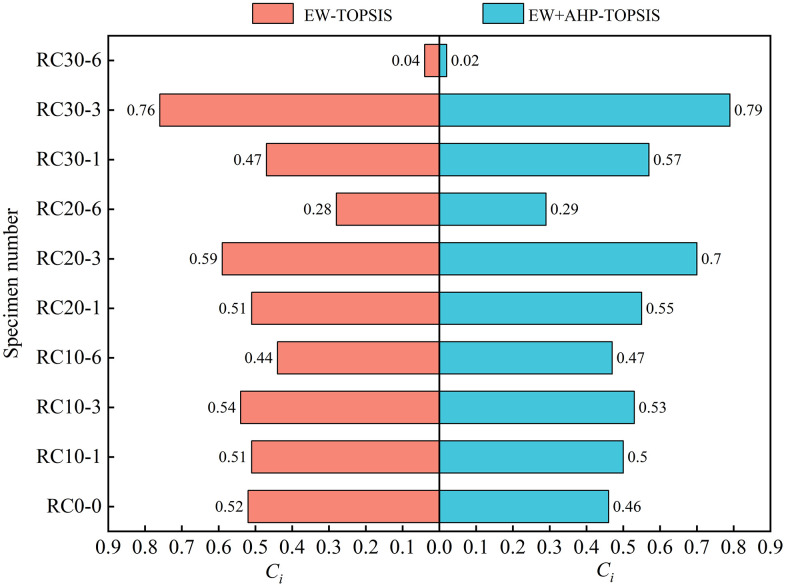
TOPSIS proximity index of the RAC scheme.

Subsequently, the Euclidean distances (*S*_*EW*_^*+*^ and *S*_*EW*_^*-*^) from each alternative in the decision matrix to the Positive Vertical Eigenvector Weight (*V*_*EW*_^*+*^) and Negative Vertical Eigenvector Weight (*V*_*EW*_^*-*^) are computed, quantifying the relative positions of the alternatives within the solution space. Based on these distances, the relative closeness coefficients are derived. The comprehensive results are illustrated in Fig 16.


$$\begin{array}{c} {S_{EW}}^{+}=\left[\begin{array}{cccccccccc} @cccccccccc@0.26 & 0.25 & 0.22 & 0.27 & 0.24 & 0.22 & 0.35 & 0.27 & 0.12 & 0.45 \end{array}\right] \end{array}$$

$$\begin{array}{c} {S_{EW}}^{-}=\left[\begin{array}{cccccccccc} @cccccccccc@0.29 & 0.26 & 0.26 & 0.22 & 0.25 & 0.31 & 0.14 & 0.24 & 0.37 & 0.02 \end{array}\right] \end{array}$$

### 4.2. Improved TOPSIS model integrating EW and AHP

#### 4.2.1. Computational procedures of the EW + AHP – TOPSIS model.

However, relying solely on the EW – TOPSIS method, which depends entirely on the inherent data structure, may overlook the intrinsic importance of certain indicators in engineering practice. To address this limitation, this paper introduces an improvement by incorporating the Analytic Hierarchy Process (AHP) [47]. AHP systematically structures and quantifies expert judgment, thereby deriving subjective weights that reflect the practical requirements of engineering applications. Ultimately, the subjective weights obtained via AHP are scientifically integrated with the objective weights derived from the EW to collectively serve as the weighting basis for the TOPSIS analysis. This enhancement ensures that the model not only adheres to the patterns present in objective data but also incorporates professional expertise, thereby improving the engineering rationality and decision – making reliability of the solution prioritization outcomes. The computational steps are as follows:

(1) AHP Weight Determination. In this study, the subjective weights for AHP were determined through consultations with five domain experts, comprising two specialists in building materials and concrete durability, one in structural engineering and life – cycle design, one in engineering economics and project management, and one in engineering practices specific to saline soil regions. Using the 1–9 scale method, pairwise comparisons were conducted for the evaluation indicators – compressive strength, electrical flux, non – harmful porosity, slightly harmful porosity, harmful porosity, and highly harmful porosity – to construct a comparison matrix M. Subsequently, the eigenvector method was employed to determine the weights, followed by a consistency check, resulting in the weight matrix W_2_ = w_2j_, as presented in Eq. (13).

$$\begin{array}{c} w_{2j}=\frac{k_{j}}{\sum_{i=1}^{m}k_{j}} \end{array}$$ (13)

Where *k*_*j*_ represents the geometric mean of each indicator.

(2) The comprehensive weighting matrix *W* = *w*_*j*_ is determined as shown in Eq. (14).

$$\begin{array}{c} w_{j}=\alpha \cdot w_{1j}+\beta \cdot w_{2j} \end{array}$$ (14)

In the equation: the study posits that subjective and objective weights are of equal importance, setting *α* = *β* = 0.5.

(3) Integrating the composite weights into the TOPSIS method. A decision matrix *V* = *v*_*ij*_ is constructed as shown in Eq. (15).

$$\begin{array}{c} v_{ij}=w_{j}\cdot y_{ij} \end{array}$$ (15)

(4) Determination of relative closeness. The positive and negative ideal solutions, along with the Euclidean distances, are calculated using Eqs. (8) to (11). Subsequently, the relative closeness is determined by applying Eq. (12). A higher value of *C*_*i*_ indicates superior chloride ion erosion resistance for the concrete mixture.
(5) Model Sensitivity Validation. To further validate the stability of the EW and AHP – enhanced TOPSIS model, a weight sensitivity analysis was conducted to address the limitations of relying solely on entropy weight – based validation. The validation process was designed by integrating the model’s characteristics with practical engineering requirements, employing sensitivity analysis. By adjusting the subjective – objective weighting coefficient α (set to 0.3, 0.5, and 0.7, respectively), the relative closeness rankings of alternative solutions under each scenario were computed.

#### 4.2.2. Optimal selection of anti-chloride ion erosion performance schemes based on the EW + AHP – TOPSIS model.

With reference to expert recommendations and the recommended weights of each evaluation criterion obtained through entropy weighting method, a comparative matrix *M* is established [48].


$$\begin{array}{c} M=\left[\begin{array}{cccccc} @cccccc@1 & 1/4 & 3 & 2 & 1/2 & 1/3\\ 4 & 1 & 6 & 5 & 3 & 2\\ 1/3 & 1/6 & 1 & 1/2 & 1/4 & 1/5\\ 1/2 & 1/5 & 2 & 1 & 1/3 & 1/4\\ 2 & 1/3 & 4 & 3 & 1 & 1/2\\ 3 & 1/2 & 5 & 4 & 2 & 1 \end{array}\right] \end{array}$$

Subsequently, the weights for each indicator are calculated to form *W*_2_.


$$\begin{array}{c} W_{\text{2}}=\left[\begin{array}{cccccc} @cccccc@0.11 & 0.37 & 0.05 & 0.07 & 0.16 & 0.24 \end{array}\right] \end{array}$$

Based on the obtained weights, a consistency check was performed on the judgment matrix *M*, with the results summarized in Table 6.

**Table 6:** Consistency check results.

Judgment matrix	λ_max_	CI	RI	CR	Consistency test
*M*	6.039	0.008	1.24	0.006	Yes

A weight matrix *W* is constructed by calculating the weights of individual indicators.


$$\begin{array}{c} W=\left[\begin{array}{cccccc} @cccccc@0.11 & 0.34 & 0.13 & 0.10 & 0.13 & 0.19 \end{array}\right] \end{array}$$

The normalized decision matrix *Y* is subjected to a dot product with the weight matrix *W*, thereby constructing a weighted normalized decision matrix *V*_*EW+AHP*_ that integrates objective weights into the decision – making framework.


$$\begin{array}{c} V_{EW+AHP}=\left[\begin{array}{cccccc} @cccccc@0.08 & 0.10 & 0.13 & 0.10 & 0.09 & 0.07\\ 0.09 & 0.10 & 0.10 & 0.09 & 0.10 & 0.13\\ 0.10 & 0.22 & 0.07 & 0.03 & 0.07 & 0.04\\ 0.09 & 0.11 & 0.06 & 0.05 & 0.08 & 0.14\\ 0.11 & 0.15 & 0.05 & 0.05 & 0.10 & 0.14\\ 0.04 & 0.28 & 0.01 & 0.04 & 0.13 & 0.19\\ 0.03 & 0.05 & 0.04 & 0.06 & 0.07 & 0.08\\ 0.07 & 0.21 & 0.00 & 0.03 & 0.10 & 0.13\\ 0.06 & 0.34 & 0.07 & 0.08 & 0.08 & 0.14\\ 0.00 & 0.00 & 0.01 & 0.00 & 0.00 & 0.00 \end{array}\right] \end{array}$$

Subsequently, the positive ideal solution (*V*_*EW+AHP*_^*+*^) and negative ideal solution (*V*_*EW+AHP*_^*-*^) are identified from the matrix *V*_*EW+AHP*_.


$$\begin{array}{c} {V_{EW+AHP}}^{+}=\left[\begin{array}{cccccc} @cccccc@0.11 & 0.34 & 0.13 & 0.10 & 0.13 & 0.19 \end{array}\right] \end{array}$$

$$\begin{array}{c} {V_{EW+AHP}}^{-}=\left[\begin{array}{cccccc} @cccccc@0.00 & 0.00 & 0.00 & 0.00 & 0.00 & 0.00 \end{array}\right] \end{array}$$

Finally, relative closeness metrics were derived for each alternative from both *S*_*EW+AHP*_^*+*^ and *S*_*EW+AHP*_^*-*^ frameworks, leading to the recommendation of the optimal combination as illustrated in Fig 16.


$$\begin{array}{c} {S_{EW+AHP}}^{+}=\left[\begin{array}{cccccccccc} @cccccccccc@0.28 & 0.25 & 0.23 & 0.26 & 0.22 & 0.16 & 0.34 & 0.21 & 0.11 & 0.45 \end{array}\right] \end{array}$$

$$\begin{array}{c} {S_{EW+AHP}}^{-}=\left[\begin{array}{cccccccccc} @cccccccccc@0.24 & 0.25 & 0.26 & 0.23 & 0.27 & 0.37 & 0.14 & 0.28 & 0.40 & 0.01 \end{array}\right] \end{array}$$

As illustrated in Fig 16, the EW – TOPSIS method and the improved EW + AHP – TOPSIS method yield overall consistent trends in the results. Both approaches confirm that solution RC30–3 (with 3% CLDHs content and 30% RA replacement rate) exhibits the optimal resistance to chloride ion erosion, while RC30–6 (with 6% CLDHs content and 30% RA replacement rate) demonstrates the poorest performance in this regard, which aligns with the experimental findings. However, there are numerical discrepancies between the two methods. In most cases, the closeness degrees obtained via the EW + AHP – TOPSIS method are more polarized. Specifically, the closeness degree of RC30–3 increases from 0.76 to 0.79, and that of RC20–3 rises from 0.59 to 0.70. Conversely, for certain groups such as RC0–0 and RC10–1, the closeness degrees slightly decrease under the EW + AHP – TOPSIS method. These observations indicate that the adjustment of weights enhances the discrimination capability of the evaluation outcomes.

Fig 16 reveals that the results obtained from the EW-TOPSIS method and the EW + AHP – TOPSIS method consistently validate scheme RC30–3 (with 3% CLDHs content and 30% RA replacement rate) as exhibiting optimal resistance to chloride ion ingress, while scheme RC30–6 (with 6% CLDHs content and 30% RA replacement rate) demonstrates the poorest performance – findings that align with the experimental outcomes. However, notable numerical discrepancies are observed between the two methods. Under the EW + AHP – TOPSIS approach, the closeness degrees for most specimen groups become more polarized. For instance, the closeness of RC30–3 rises from 0.76 to 0.79, and that of RC20–3 increases from 0.59 to 0.70. Conversely, the closeness of some groups, such as RC0–0 and RC10–1, diminishes under the EW + AHP – TOPSIS method. This indicates that the adjusted weighting scheme enhances the discriminative capacity of the evaluation results.

To further validate the improved TOPSIS model integrating EW + AHP, sensitivity analysis was conducted to assess the chloride ion penetration resistance of RAC under varying α values. When AHP + EW combination weights assigned β (representing the AHP weighting factor) as 0.5, 0.7, or 0.9 – indicating dominance of AHP or equal influence of both factors – the ranking of RAC specimens remained consistent, specifically: RC30–3 > RC20–3 > RC30–1 > RC20–1 > RC10–3 > RC10–1 > RC10–6 > RC0–0 > RC20–6 > RC30–6. Conversely, when α was set to 0.5, 0.7, or 0.9 – reflecting EW dominance or balanced weighting – the rankings showed variability. For example, with α = 0.5, specimen RC10–3 ranked fifth, whereas with α = 0.7, it ranked third. These results demonstrate that the rankings produced by the AHP + EW – TOPSIS model remain stable when AHP either predominates or shares equal weight, which markedly exceeds the ranking consistency achievable with the traditional EW – TOPSIS model, thereby confirming the superior robustness of the improved model.

### 4.3. Economic feasibility analysis model

Based on the optimized chloride ion corrosion resistance performance scheme, this study evaluates the cost-effectiveness of RAC and provides data support for selecting optimal construction materials and building strategies. The assessment primarily encompasses upstream processes including raw material acquisition, material transportation, and concrete preparation. The total cost per cubic meter of concrete is calculated using Eq (16), with computational outcomes presented in Table 5. For each material category, comprehensive cost components are considered, covering initial procurement price, transportation expenses, energy consumption, equipment depreciation, and yield loss costs [49].


$$\begin{array}{c} C=\sum_{i=1}^{\text{7}}C_{i} \end{array}$$ (16)

Where *C*_*i*_ denotes the cost of material *i*.

Table 7 indicates that CLDHs contribute dominantly to cost escalation, while RA alleviates the cost burden. Compared to RC20–1, which achieved the optimal compressive strength, RC30–3 – exhibiting the best chloride ion penetration resistance – incurred an additional cost of 16.2 USD/m³, representing an increase of approximately 17%. Although CLDHs carry a relatively high unit price, their incorporation rate ranges only from 1% to 6%, resulting in a limited cost increment per cubic meter of concrete. More importantly, CLDHs significantly enhance the chloride erosion resistance of RAC, extend the service life of structures in saline soil environments, and reduce lifecycle maintenance costs. For moderate to severe corrosive environments such as coastal and saline – alkali regions, this cost increment is acceptable, thereby demonstrating practical engineering applicability. However, CLDHs - modified concrete is not recommended for non – corrosive environments or temporary structures.

**Table 7:** Economic assessment of the radial artery craft.

Specimen NO.	Cost/USD·m^-3^							Cost/USD·m^-3^
	OPC	CLDHs	Water	River sand	Crushed stone	Recycled aggregate	water reduce	
RC0−0	30.6	0	0.0	1.1	16.5	0	41.3	89.5
RC10−1	30.3	9.3	0.0	1.1	14.8	0.3	41.3	97.1
RC10−3	29.6	27.5	0.0	1.1	14.8	0.3	41.3	114.6
RC10−6	28.7	55.0	0.0	1.1	14.8	0.3	41.3	141.2
RC20−1	30.3	9.2	0.0	1.1	13.2	0.6	41.3	95.7
RC20−3	29.6	27.5	0.0	1.1	13.2	0.6	41.3	113.3
RC20−6	28.7	55.0	0.0	1.1	13.2	0.6	41.3	139.9
RC30−1	30.3	9.2	0.0	1.1	11.5	0.9	41.3	94.3
RC30−3	29.6	27.5	0.0	1.1	11.5	0.9	41.3	111.9
RC30−6	28.7	55.0	0.0	1.1	11.5	0.9	41.3	138.5

In summary, if ranked solely by a single parameter, RC20–1 yields the optimal compressive strength, whereas RC30–3 ranks highest when considering only electrical flux. These conflicting outcomes based on distinct performance indicators pose challenges for engineering decision – making. To address this issue, the EW + AHP – TOPSIS model proposed in this study integrates six criteria – including compressive strength, electrical flux, and multi – scale pore structure characteristics – by combining subjective and objective weighting methods. The approach further incorporates economic considerations to provide a flexible decision – support framework for selecting mix proportions under varying corrosion environments, as illustrated in Table 8.

**Table 8:** Recommended decision alternatives.

Engineering context	Priority metrics	Recommended formulation ratio
Non-corrosive environment	High intensity prioritization	RC20−1
Moderate corrosive environment	Overall balance	RC20−3
Highly corrosive environment	Priority of impermeability resistance	RC30−3

## 5. Conclusions

This study investigates the effects of varying CLDHs dosage and RA replacement ratio on the mechanical properties, chloride ion penetration resistance, and microstructure of RAC through a series of experimental approaches, including compressive strength tests, chloride ion (Cl^-^) penetration tests, XRD, SEM, NMR. The synergistic mechanism between CLDHs and RA is elucidated, and an evaluation model for chloride ion ingress resistance is established. The principal findings are summarized as follows:

(1) The synergistic interaction between an optimal dosage of CLDHs and RA significantly enhances the mechanical properties of RAC. Compared to the control group RC0–0, the 28 d compressive strength of the RC20–1 mixture (with 20% RA replacement rate and 1% CLDHs addition) increased by 10.3% (from 54.1 MPa for RC0–0 to 59.8 MPa for RC20–1). The underlying mechanism involves the effective filling of microscopic pores within the concrete matrix by CLDHs particles, coupled with the interlocking effect between the complex surface morphology of RA and CLDHs particles, which optimizes the continuity of particle gradation. However, excessive incorporation of CLDHs or an imbalanced ratio with RA disrupts the compatibility equilibrium, leading to a decline in strength.
(2) The CLDHs significantly enhance the chloride ion penetration resistance of RAC. Leveraging both the nano – filling effect and unique layered structure, CLDHs refine pore structures and impede chloride ion transport pathways. Moreover, they immobilize chloride ions introduced by RA through physical adsorption and ion exchange, thereby mitigating steel corrosion at the source. Among the tested formulations, the RC30–3 group (with 30% RA substitution and 3% CLDHs dosage) exhibits the optimal anti – permeability performance, achieving the lowest electrical flux of 1135 C (compared to 1403 C for the RC0–0 reference group), which corresponds to a 19.1% improvement in chloride ion penetration resistance.
(3) Microstructural analysis reveals the hydration mechanism and pore structure evolution of RAC: At 28 d of curing, the hydration reaction was fully developed, with extensive formation of C – S – H gel and a marked reduction in the diffraction peak intensity of Ca(OH)_2_. The RC20–1 mixture was identified as reaching the “golden equilibrium point” of hydration. SEM observations confirmed that an appropriate amount of CLDHs promotes the formation of a continuous and compact flocculated C – S – H gel, whereas excessive CLDHs result in increased porosity and a more porous microstructure. Pore structure analysis via NMR further indicated that a 30% replacement ratio of RA yielded the least number of large pores. The incorporation of 1% CLDHs significantly increased the proportion of gel pores and transition pores; however, an excessive dosage of 6% CLDHs disrupted the pore balance, leading to an elevated proportion of harmful pores and enhanced connectivity, ultimately causing a simultaneous deterioration in mechanical properties and permeability resistance.This study did not independently conduct chloride ion adsorption isotherm experiments, as this aspect will be a key focus in subsequent research.
(4) An enhanced TOPSIS model integrating EW and AHP is proposed to systematically evaluate the chloride ion erosion resistance of RAC. Concurrently, an economic feasibility analysis is introduced to establish an adjustable decision – making framework for selecting RAC mix proportions under varying corrosion conditions. For non – corrosive, moderate corrosive, and severe corrosive environments, mix ratios RC20–1, RC20–3, and RC30–3 are recommended, respectively. This approach overcomes the limitations of single weighting methods, significantly improves the scientific rigor and engineering applicability of decision – making, and provides a reliable basis for optimizing the mix design of recycled concrete in aggressive environments.

In summary, this study preliminarily clarifies the synergistic mechanism between CLDHs and RA in enhancing the resistance of RAC to chloride ion penetration. By integrating microscopic analyses and macroscopic performance evaluation, it reveals the pore structure optimization characteristics imparted by CLDHs on RA. An improved TOPSIS evaluation model combining subjective and objective dimensions is established, enabling a comprehensive multi – criteria assessment of the synergistic effect of CLDHs and RA on improving the chloride ion penetration resistance of RAC. This work advances the mix proportion optimization methodology for such concrete and provides adjustable decision – making references for mix proportion selection under varying corrosion environments. The microscopic analysis demonstrates that the optimization of hydration products and refinement of pore structure are critical factors underpinning the macroscopic performance enhancement. It should be noted that this study employs only the DTL-9T rapid chloride permeability test as the macroscopic evaluation indicator for chloride ion penetration resistance. While this method rapidly reflects concrete's resistance to chloride ion migration, it does not fully capture the entire process of chloride adsorption, binding, and diffusion within concrete. Subsequent research will incorporate metrics such as chloride binding capacity and diffusion coefficients to refine the durability evaluation framework. Furthermore, early – age strength optimization strategies and the mechanism by which CLDHs influence the interfacial transition zone of RA remain key directions for future investigation.

## Supporting information

S1 Fig Fig.S1 shows the scene of experimental personnel performing laboratory operations. (PNG)
