# Supplementary figures and images for: Study on chloride ion erosion resistance of recycled aggregate concrete based on an improved TOPSIS model integrating entropy weight and AHP
Source: PLoS One. 2026 Jul 22;21(7):e0352439. doi: 10.1371/journal.pone.0352439 (PMC13390877; doi:10.1371/journal.pone.0352439)

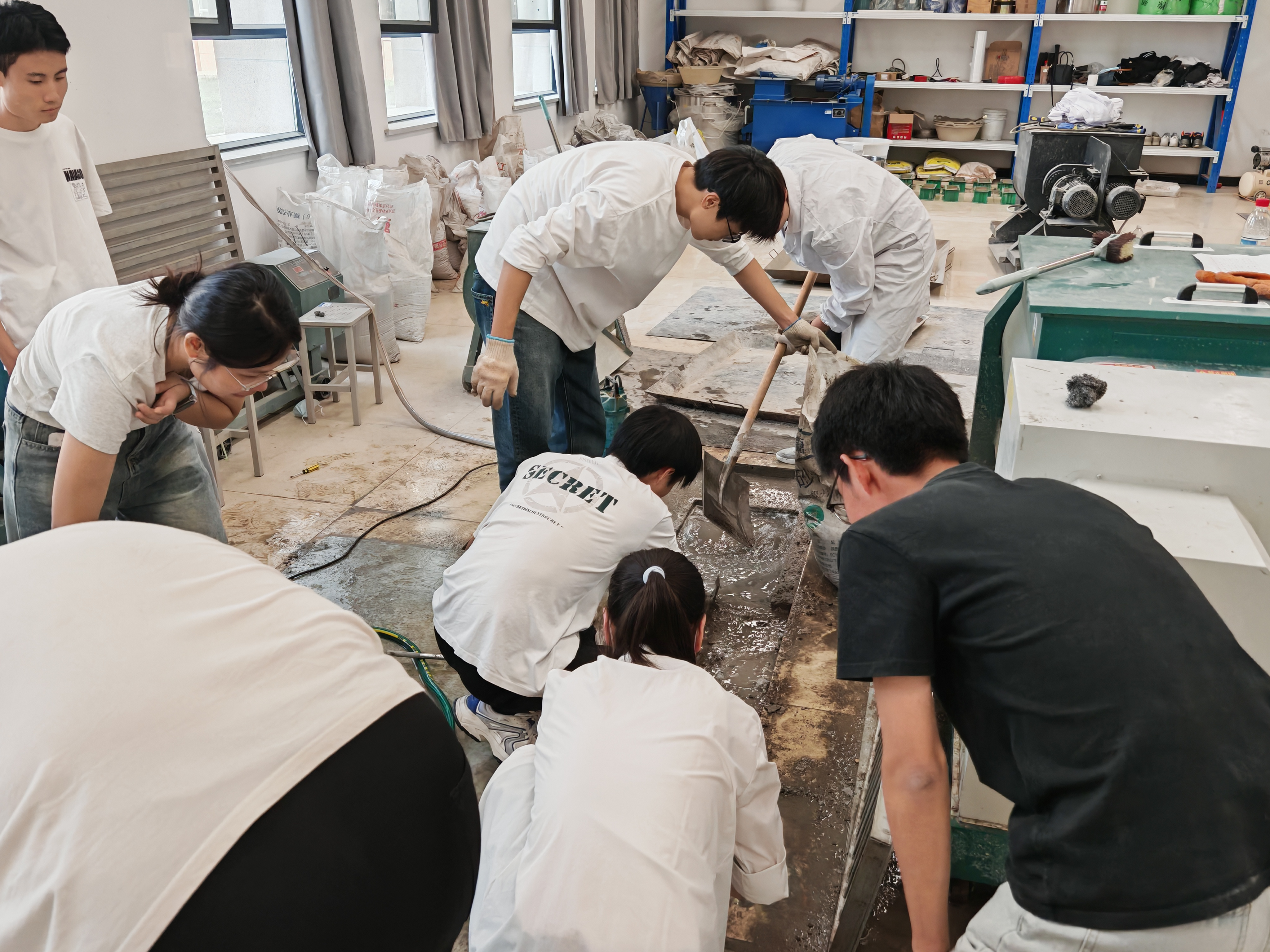

Supplement: S1 Fig — (PNG) [file pone.0352439.s001.png]
