# Supplementary material for: Study on chloride ion erosion resistance of recycled aggregate concrete based on an improved TOPSIS model integrating entropy weight and AHP
Source: PLoS One. 2026 Jul 22;21(7):e0352439. doi: 10.1371/journal.pone.0352439 (PMC13390877; doi:10.1371/journal.pone.0352439)
Supplement: Attachment — Submitted filename: PONE-D-26-05199_Technical_Queries_Table.pdf [file pone.0352439.s003.pdf]

Question No.	Question Address			Question Text
	Title	Line Number	Keyword	
**1**	Abstract & Results	40-44 332-335	Synergy	The abstract and results state that the mix with 1% CLDHs and 20% RA yielded the best compressive strength, while 3% CLDHs and 30% RA was optimal for chloride resistance. Please explain how an engineering designer should balance these two objectives (mechanical strength vs. permeability) in practical applications?
**2**	2.1. Raw Materials	139	500 °C	Considering the use of Recycled Aggregates (RA) and Calcined Layered Double Hydroxides (CLDHs), please provide a brief analysis of the economic feasibility of this approach. Does the cost associated with the calcination process of hydrotalcite at 500°C outweigh the long-term technical benefits, such as enhanced concrete durability?
**3**	2.3.1. Compressive strength tests	194-196	One group of three cubes (repetition)	In section 2.3.1, it is mentioned that the average of three cubic specimens was used for each compressive strength group. Was a similar repetition procedure followed for other tests, such as chloride ion penetration and NMR? Specifying the exact number of tested specimens for each section would enhance the paper's scientific rigor.
**4**	3.1.1. Effects of CLDHs and RA on compressive strength of concrete	270-277	At 3 d, the compressive strength ...	In the 3-day compressive strength results, it was observed that the strength of all groups was lower than the control group. The author attributes this to water absorption by CLDHs and defects in the Interface Transition Zone (ITZ). Does the author suggest any strategy to improve early-age strength in this type of concrete, such as the use of accelerating admixtures?
**5**	4.2.1. Computational procedures of the EW + AHP - TOPSIS model	627-635	TOPSIS	The author utilized an improved TOPSIS model integrating Entropy Weight (EW) and Analytic Hierarchy Process (AHP). Please clarify how many expert opinions were considered for the subjective weighting (AHP) or what standards were followed to ensure the model's reliability across different engineering environments?
