# Supplementary material for: Study on chloride ion erosion resistance of recycled aggregate concrete based on an improved TOPSIS model integrating entropy weight and AHP
Source: PLoS One. 2026 Jul 22;21(7):e0352439. doi: 10.1371/journal.pone.0352439 (PMC13390877; doi:10.1371/journal.pone.0352439)
Supplement: Attachment — Submitted filename: PONE-D-26-05199_Cover_Letter_to_Editor.pdf [file pone.0352439.s004.pdf]

Dear Editor-in-Chief of PLOS ONE,

I would like to sincerely thank you for inviting me to review the manuscript titled "**Study on Chloride Ion Erosion Resistance of Recycled Aggregate Concrete Based on an Improved TOPSIS Model Integrating Entropy Weight and AHP**" (Manuscript No. **PONE-D-26-05199**).

I have carefully reviewed the manuscript and provided my technical comments. To facilitate the revision process for the authors and the editorial team, my inquiries and suggestions have been organized into two formats:

1. Direct Annotations in the PDF: I have highlighted the relevant parts within the manuscript and added corresponding comments for each section.

2. Detailed Summary Table: I have prepared a separate PDF file containing a table that includes the question numbers along with precise location details for each query, such as the section title, line number, and keywords. This ensures there is no ambiguity regarding the context of each question.

In my professional assessment, this work is of significant value. The authors have demonstrated a robust methodology, particularly in the integration of advanced techniques like NMR and SEM with the EW+AHP-TOPSIS decision-making model. The research addresses a crucial gap in the use of recycled aggregates and calcined layered double hydroxides (CLDHs).

While the study is well-executed, I believe it requires **Minor Revisions** to further enhance its clarity and impact, especially regarding the economic feasibility and certain technical clarifications. Once these points are addressed, I believe this valuable work will be highly suitable for publication in PLOS ONE.

I look forward to further collaborations with **PLOS ONE** in the future.

Sincerely,

Reviewer of PONE-D-26-05199
