# Supplementary material for: Study on chloride ion erosion resistance of recycled aggregate concrete based on an improved TOPSIS model integrating entropy weight and AHP
Source: PLoS One. 2026 Jul 22;21(7):e0352439. doi: 10.1371/journal.pone.0352439 (PMC13390877; doi:10.1371/journal.pone.0352439)
Supplement: Attachment — Submitted filename: Responses to Reviewer s Comments PONE-D-26-05199.doc [file pone.0352439.s006.doc]

**Study on chloride ion erosion resistance of recycled aggregate concrete based on an improved TOPSIS model integrating entropy weight and AHP**

**Manuscript Number**: PONE-D-26-05199

**Authors:** Yali Cao , Wenbang Zhu , Xinjie Wang , Chuikan Li , Ruiming Liu ，Dali Zhang , Xiumei Zheng , BaochenTang

The author team would like to express our heartfelt gratitude to the editor and reviewers for the comprehensive, in-depth, and constructive review of our manuscript. We sincerely appreciate the insightful comments provided by each reviewer. These comments are not only essential for the revision and refinement of this paper but also hold great significance in guiding our subsequent research endeavors, thereby providing crucial support for elevating the quality of our work and deepening the research.

In response to the reviewers’ comments, we have completed a series of responses and revisions. We have provided detailed replies to each comment from every reviewer. Additionally, we have not only marked the revised sections of the paper in red but also conducted a thorough revision of the entire manuscript to ensure its academic rigor, logical coherence, and overall integrity.

For your and the reviewers’ convenience, the original comments are enclosed in boxes within this document, and our specific responses are presented in italics for quick reference. Correspondingly, all modifications and supplementary content in the manuscript are uniformly highlighted in red to make the revisions evident.

We have made every effort to ensure all revisions are clearly presented. We earnestly hope that the revised paper meets the publication criteria of your esteemed journal and contributes to the enhancement of its academic value. Once again, we extend our sincere appreciation to you and the reviewers for your professional support and guidance throughout this research.

Journal Requirements:

When submitting your revision, we need you to address these additional requirements.

1. Please ensure that your manuscript meets PLOS ONE's style requirements, including those for file naming. The PLOS ONE style templates can be found at

https://journals.plos.org/plosone/s/file?id=wjVg/PLOSOne_formatting_sample_main_body.pdf and

https://journals.plos.org/plosone/s/file?id=ba62/PLOSOne_formatting_sample_title_authors_affiliations.pdf.

*Response:We sincerely appreciate the reminders and guidance provided by the editorial team. We have conducted a comprehensive review and revision of the manuscript in strict accordance with PLOS ONE’s formatting requirements, including proper file naming protocols, to ensure full compliance with the journal’s submission standards. Thank you once again for your attentive support.*

2. We note that this submission includes NMR spectroscopy data. We would recommend that you include the following information in your methods section or as Supporting Information files:

1) The make/source of the NMR instrument used in your study, as well as the magnetic field strength. For each individual experiment, please also list: the nucleus being measured; the sample concentration; the solvent in which the sample is dissolved and if solvent signal suppression was used; the reference standard and the temperature.

2) A list of the chemical shifts for all compounds characterised by NMR spectroscopy, specifying, where relevant: the chemical shift (δ), the multiplicity and the coupling constants (in Hz), for the appropriate nuclei used for assignment.

3)The full integrated NMR spectrum, clearly labelled with the compound name and chemical structure.

We also strongly encourage authors to provide primary NMR data files, in particular for new compounds which have not been characterised in the existing literature. Authors should provide the acquisition data, FID files and processing parameters for each experiment, clearly labelled with the compound name and identifier, as well as a structure file for each provided dataset. See our list of recommended repositories here: https://journals.plos.org/plosone/s/recommended-repositories

*Response:We would like to express our sincere gratitude to the editorial team for their professional guidance and meticulous reminders. It is important to clarify that the nuclear magnetic resonance (NMR) analysis employed in this study was exclusively utilized for characterizing the pore structure of concrete materials and not for compound identification. As such, no chemical shift data, coupling constants, raw FID data, or structural files of compounds are involved. We have now comprehensively supplemented the methodology section with detailed information regarding the NMR instrument model, manufacturer, testing conditions, and key experimental parameters. Furthermore, clearly annotated spectra and corresponding analyses have been provided in the main text, ensuring full compliance with the characterization standards in the field of materials science as well as the journal's requirements. We sincerely appreciate the editorial team's attentive guidance once again.*

3. PLOS requires an ORCID iD for the corresponding author in Editorial Manager on papers submitted after December 6th, 2016. Please ensure that you have an ORCID iD and that it is validated in Editorial Manager. To do this, go to ‘Update my Information’ (in the upper left-hand corner of the main menu), and click on the Fetch/Validate link next to the ORCID field. This will take you to the ORCID site and allow you to create a new iD or authenticate a pre-existing iD in Editorial Manager.

*Response:We sincerely appreciate the meticulous reminders and professional guidance provided by the editorial team. We have fully acknowledged the journal's requirement that corresponding authors must provide and validate their ORCID iD. Accordingly, we have now completed the registration and validation of the corresponding author's ORCID iD within the Editorial Manager system, ensuring that the information is accurate, valid, and synchronized with the system. Once again, we extend our heartfelt gratitude for the editorial team’s attentive support and assistance.*

4. We note that the grant information you provided in the ‘Funding Information’ and ‘Financial Disclosure’ sections do not match.

When you resubmit, please ensure that you provide the correct grant numbers for the awards you received for your study in the ‘Funding Information’ section.

*Response:We sincerely appreciate the meticulous reminders and professional guidance provided by the editorial team. We have thoroughly reviewed and aligned the funding information in both the "Funding" and "Financial Disclosure" sections to ensure complete consistency. The accurate project number associated with this study has been correctly entered in the funding section. All relevant details have been meticulously revised to fully comply with the journal’s requirements. Once again, we extend our heartfelt gratitude to the editorial team for their invaluable support and guidance.*

5. Thank you for stating the following financial disclosure:

The author(s) received no specific funding for this work.

At this time, please address the following queries:

a) Please clarify the sources of funding (financial or material support) for your study. List the grants or organizations that supported your study, including funding received from your institution.

b) State what role the funders took in the study. If the funders had no role in your study, please state: “The funders had no role in study design, data collection and analysis, decision to publish, or preparation of the manuscript.”

c) If any authors received a salary from any of your funders, please state which authors and which funders.

d) If you did not receive any funding for this study, please state: “The authors received no specific funding for this work.”

Please include your amended statements within your cover letter; we will change the online submission form on your behalf.

*Response:We express our sincere gratitude to the editorial team for their meticulous review and valuable guidance. This study was supported by the Natural Science Foundation of the Xinjiang Uygur Autonomous Region (Grant No. 2025D01A05), the National Innovation and Entrepreneurship Training Program for College Students of China (Grant No. 202510763009), and the Innovation and Entrepreneurship Training Program for College Students of the Xinjiang Uygur Autonomous Region (Grant No. S202510763042). The funding bodies played no role in the study design, data collection and analysis, decision to publish, or preparation of the manuscript. None of the authors received salaries or other forms of remuneration from any funding organization in relation to this research. The relevant financial disclosure statement has been updated as required and is available for review.*

6. Thank you for stating the following in the Acknowledgments Section of your manuscript:

This research was funded by the Xinjiang Uygur Autonomous Region Natural Science Foundation (No. 2025D01A05), the China College Students’ Innovation and Entrepreneurship Development Program (No. 202510763009), and the Xinjiang UygurAutonomous Region College Students’ Innovation and Entrepreneurship Training Program (No. S202510763042).

We note that you have provided funding information that is not currently declared in your Funding Statement. However, funding information should not appear in the Acknowledgments section or other areas of your manuscript. We will only publish funding information present in the Funding Statement section of the online submission form.

Please remove any funding-related text from the manuscript and let us know how you would like to update your Funding Statement. Currently, your Funding Statement reads as follows:

The author(s) received no specific funding for this work.

Please include your amended statements within your cover letter; we will change the online submission form on your behalf.

*Response:We sincerely appreciate the editor's prompt notification. In compliance with the request, all funding-related content has been removed from both the main text and acknowledgments section of the manuscript, and the funding disclosure statement in the submission system will be updated accordingly. The revised funding statement is as follows:*

*This research was supported by the Natural Science Foundation of Xinjiang Uygur Autonomous Region (Grant No. 2025D01A05), the National Innovation and Entrepreneurship Training Program for College Students in China (Grant No. 202510763009), and the Xinjiang Uygur Autonomous Region Innovation and Entrepreneurship Training Program for College Students (Grant No. S202510763042). The funders had no involvement in the study design, data collection and analysis, decision to publish, or manuscript preparation. None of the authors received salaries or other forms of compensation related to this research from any of the above-mentioned funders.*

7. We note that your Data Availability Statement is currently as follows: All relevant data are within the manuscript and its Supporting Information files.

Please confirm at this time whether or not your submission contains all raw data required to replicate the results of your study. Authors must share the “minimal data set” for their submission. PLOS defines the minimal data set to consist of the data required to replicate all study findings reported in the article, as well as related metadata and methods (https://journals.plos.org/plosone/s/data-availability#loc-minimal-data-set-definition).

For example, authors should submit the following data:

- The values behind the means, standard deviations and other measures reported;

- The values used to build graphs;

- The points extracted from images for analysis.

Authors do not need to submit their entire data set if only a portion of the data was used in the reported study.

If your submission does not contain these data, please either upload them as Supporting Information files or deposit them to a stable, public repository and provide us with the relevant URLs, DOIs, or accession numbers. For a list of recommended repositories, please see https://journals.plos.org/plosone/s/recommended-repositories.

If there are ethical or legal restrictions on sharing a de-identified data set, please explain them in detail (e.g., data contain potentially sensitive information, data are owned by a third-party organization, etc.) and who has imposed them (e.g., an ethics committee). Please also provide contact information for a data access committee, ethics committee, or other institutional body to which data requests may be sent. If data are owned by a third party, please indicate how others may request data access.

*Response: We would like to express our sincere gratitude to the editorial team for their professional reminders and meticulous guidance. The submitted manuscript for this research already contains all the minimum data sets necessary for the reproducibility of the research results. The original data, chart values, image analysis data, and related metadata and experimental methods supporting all the research conclusions of this paper have been fully provided in the main text, and there is no need to upload them to external databases. The data availability statement has been confirmed and improved in accordance with the journal requirements, ensuring compliance with the data sharing policy of PLOS ONE. Once again, we sincerely thank the editorial team for their careful guidance and assistance!*

8. Please amend your list of authors on the manuscript to ensure that each author is linked to an affiliation. Authors’ affiliations should reflect the institution where the work was done (if authors moved subsequently, you can also list the new affiliation stating “current affiliation:….” as necessary).

*Response:We acknowledge the editorial team's meticulous reminder. A thorough verification and revision of the author list have been completed, with each contributor accurately linked to their institutional affiliation at the time the research was conducted, ensuring full correspondence between the institutional details provided and the entities where the research was carried out. In instances where current affiliations differ, these have been appropriately annotated with the phrase "Current affiliation: ..." as required. The revised author and institutional details have been comprehensively updated in the manuscript to ensure full compliance with academic standards.*

9. Thank you for stating the following in the Competing Interests/Financial Disclosure section:

The authors have declared that no competing interests exist.

We note that one or more of the authors are employed by a commercial company: Kashi Zhengxin Construction Engineering Testing Co., Ltd

a. Please provide an amended Funding Statement declaring this commercial affiliation, as well as a statement regarding the Role of Funders in your study. If the funding organization did not play a role in the study design, data collection and analysis, decision to publish, or preparation of the manuscript and only provided financial support in the form of authors' salaries and/or research materials, please review your statements relating to the author contributions, and ensure you have specifically and accurately indicated the role(s) that these authors had in your study. You can update author roles in the Author Contributions section of the online submission form.

Please also include the following statement within your amended Funding Statement.

“The funder provided support in the form of salaries for authors [insert relevant initials], but did not have any additional role in the study design, data collection and analysis, decision to publish, or preparation of the manuscript. The specific roles of these authors are articulated in the ‘author contributions’ section.”

If your commercial affiliation did play a role in your study, please state and explain this role within your updated Funding Statement.

b. Please also provide an updated Competing Interests Statement declaring this commercial affiliation along with any other relevant declarations relating to employment, consultancy, patents, products in development, or marketed products, etc.

Within your Competing Interests Statement, please confirm that this commercial affiliation does not alter your adherence to all PLOS ONE policies on sharing data and materials by including the following statement: "This does not alter our adherence to PLOS ONE policies on sharing data and materials.” (as detailed online in our guide for authors http://journals.plos.org/plosone/s/competing-interests) . If this adherence statement is not accurate and there are restrictions on sharing of data and/or materials, please state these. Please note that we cannot proceed with consideration of your article until this information has been declared.

Please include both an updated Funding Statement and Competing Interests Statement in your cover letter. We will change the online submission form on your behalf.

*Response:We extend our sincere gratitude to the editorial team for their meticulous review and professional guidance. As per requirements, the following declarations have been provided in the cover letter and Declaration of Interest Statement: Author Li Chuikan is employed by Kashi Zhengxin Construction Engineering Testing Co., Ltd. The company’s support was limited to providing the author’s salary and did not involve any additional role in the study design, data collection and analysis, decision to publish, or manuscript preparation. The specific role of this author has been elaborated in the Author Contributions section. Beyond this, no other conflicts of interest exist among the authors. Furthermore, this commercial affiliation does not affect our adherence to PLOS ONE’s policies on data and material sharing. We kindly request your review.*

10. If the reviewer comments include a recommendation to cite specific previously published works, please review and evaluate these publications to determine whether they are relevant and should be cited. There is no requirement to cite these works unless the editor has indicated otherwise.

*Response:Thank you for the editor’s reminder. The current review comments do not indicate any specific references that require inclusion; therefore, no supplementary amendments to the reference section are necessary. The relevant content has been verified as requested and is now submitted for review.*

Reviewer #1:

The manuscript is scientifically well-constructed. If the author provides a convincing explanation regarding the economic feasibility (as requested in the comments) and clarifies the weighting model, the paper will be highly valuable for the field. I recommend Minor Revision leading toward acceptance.

*Response1:We sincerely appreciate the reviewers' valuable comments. In the revised manuscript, we have incorporated an additional section discussing the cost and engineering feasibility of CLDHs.****(See the revised draft lines 744-783)***

4.3. Economic feasibility analysis model

Based on the optimized chloride ion corrosion resistance performance scheme, this study evaluates the cost-effectiveness of RAC and provides data support for selecting optimal construction materials and building strategies. The assessment primarily encompasses upstream processes including raw material acquisition, material transportation, and concrete preparation. The total cost per cubic meter of concrete is calculated using Eq (16), with computational outcomes presented in Table 5. For each material category, comprehensive cost components are considered, covering initial procurement price, transportation expenses, energy consumption, equipment depreciation, and yield loss costs [49].

| $C=\sum_{i=1}^{7} C_{i}$ | （16） |
| --- | --- |

Where *C_i_* denotes the cost of material *i*.

**Table 7. Economic assessment of the radial artery craft**

| Specimen NO. | Cost/USD·m^-3^ | | | | | | | Cost/USD·m^-3^ |
| --- | --- | --- | --- | --- | --- | --- | --- | --- |
|  | OPC | CLDHs | Water | River sand | Crushed  stone | Recycled aggregate | water reduce |  |
| RC0-0 | 30.6 | 0 | 0.0 | 1.1 | 16.5 | 0 | 41.3 | 89.5 |
| RC10-1 | 30.3 | 9.3 | 0.0 | 1.1 | 14.8 | 0.3 | 41.3 | 97.1 |
| RC10-3 | 29.6 | 27.5 | 0.0 | 1.1 | 14.8 | 0.3 | 41.3 | 114.6 |
| RC10-6 | 28.7 | 55.0 | 0.0 | 1.1 | 14.8 | 0.3 | 41.3 | 141.2 |
| RC20-1 | 30.3 | 9.2 | 0.0 | 1.1 | 13.2 | 0.6 | 41.3 | 95.7 |
| RC20-3 | 29.6 | 27.5 | 0.0 | 1.1 | 13.2 | 0.6 | 41.3 | 113.3 |
| RC20-6 | 28.7 | 55.0 | 0.0 | 1.1 | 13.2 | 0.6 | 41.3 | 139.9 |
| RC30-1 | 30.3 | 9.2 | 0.0 | 1.1 | 11.5 | 0.9 | 41.3 | 94.3 |
| RC30-3 | 29.6 | 27.5 | 0.0 | 1.1 | 11.5 | 0.9 | 41.3 | 111.9 |
| RC30-6 | 28.7 | 55.0 | 0.0 | 1.1 | 11.5 | 0.9 | 41.3 | 138.5 |

Table 7 indicates that CLDHs contribute dominantly to cost escalation, while RA alleviates the cost burden. Compared to RC20 - 1, which achieved the optimal compressive strength, RC30 - 3 - exhibiting the best chloride ion penetration resistance - incurred an additional cost of 16.2 USD/m³, representing an increase of approximately 17%. Although CLDHs carry a relatively high unit price, their incorporation rate ranges only from 1% to 6%, resulting in a limited cost increment per cubic meter of concrete. More importantly, CLDHs significantly enhance the chloride erosion resistance of RAC, extend the service life of structures in saline soil environments, and reduce lifecycle maintenance costs. For moderate to severe corrosive environments such as coastal and saline - alkali regions, this cost increment is acceptable, thereby demonstrating practical engineering applicability. However, CLDHs - modified concrete is not recommended for non - corrosive environments or temporary structures.

In summary, if ranked solely by a single parameter, RC20 - 1 yields the optimal compressive strength, whereas RC30 - 3 ranks highest when considering only electrical flux. These conflicting outcomes based on distinct performance indicators pose challenges for engineering decision - making. To address this issue, the EW + AHP - TOPSIS model proposed in this study integrates six criteria - including compressive strength, electrical flux, and multi - scale pore structure characteristics - by combining subjective and objective weighting methods. The approach further incorporates economic considerations to provide a flexible decision - support framework for selecting mix proportions under varying corrosion environments, as illustrated in Table 8.

**Table 8. Recommended decision alternatives**

| Engineering context | Priority metrics | Recommended formulation ratio |
| --- | --- | --- |
| Non-corrosive environment | High intensity prioritization | RC20-1 |
| Moderate corrosive environment | Overall balance | RC20-3 |
| Highly corrosive environment | Priority of impermeability resistance | RC30-3 |

*Response2:Thank you for highlighting this point. We have supplemented the following content in the section titled “Improved TOPSIS Model Incorporating Entropy Weight and AHP Method” of the revised manuscript.****(See the revised draft lines 652-662, 674-681 and 717-742)***

(1)AHP Weight Determination. In this study, the subjective weights for AHP were determined through consultations with five domain experts, comprising two specialists in building materials and concrete durability, one in structural engineering and life - cycle design, one in engineering economics and project management, and one in engineering practices specific to saline soil regions. Using the 1 - 9 scale method, pairwise comparisons were conducted for the evaluation indicators - compressive strength, electrical flux, non - harmful porosity, slightly harmful porosity, harmful porosity, and highly harmful porosity - to construct a comparison matrix M. Subsequently, the eigenvector method was employed to determine the weights, followed by a consistency check, resulting in the weight matrix W_2_ = w_2j_, as presented in Eq. (13).

(5) Model Sensitivity Validation. To further validate the stability of the EW and AHP - enhanced TOPSIS model, a weight sensitivity analysis was conducted to address the limitations of relying solely on entropy weight - based validation. The validation process was designed by integrating the model's characteristics with practical engineering requirements, employing sensitivity analysis. By adjusting the subjective - objective weighting coefficient α (set to 0.3, 0.5, and 0.7, respectively), the relative closeness rankings of alternative solutions under each scenario were computed.

Fig 16 reveals that the results obtained from the EW-TOPSIS method and the EW + AHP - TOPSIS method consistently validate scheme RC30 - 3 (with 3% CLDHs content and 30% RA replacement rate) as exhibiting optimal resistance to chloride ion ingress, while scheme RC30 - 6 (with 6% CLDHs content and 30% RA replacement rate) demonstrates the poorest performance - findings that align with the experimental outcomes. However, notable numerical discrepancies are observed between the two methods. Under the EW + AHP - TOPSIS approach, the closeness degrees for most specimen groups become more polarized. For instance, the closeness of RC30 - 3 rises from 0.76 to 0.79, and that of RC20 - 3 increases from 0.59 to 0.70. Conversely, the closeness of some groups, such as RC0 - 0 and RC10 - 1, diminishes under the EW + AHP - TOPSIS method. This indicates that the adjusted weighting scheme enhances the discriminative capacity of the evaluation results.

To further validate the improved TOPSIS model integrating EW + AHP, sensitivity analysis was conducted to assess the chloride ion penetration resistance of RAC under varying α values. When AHP + EW combination weights assigned β (representing the AHP weighting factor) as 0.5, 0.7, or 0.9 - indicating dominance of AHP or equal influence of both factors - the ranking of RAC specimens remained consistent, specifically: RC30 - 3 > RC20 - 3 > RC30 - 1 > RC20 - 1 > RC10 - 3 > RC10 - 1 > RC10 - 6 > RC0 - 0 > RC20 - 6 > RC30 - 6. Conversely, when α was set to 0.5, 0.7, or 0.9 - reflecting EW dominance or balanced weighting - the rankings showed variability. For example, with α = 0.5, specimen RC10 - 3 ranked fifth, whereas with α = 0.7, it ranked third. These results demonstrate that the rankings produced by the AHP + EW - TOPSIS model remain stable when AHP either predominates or shares equal weight, which markedly exceeds the ranking consistency achievable with the traditional EW - TOPSIS model, thereby confirming the superior robustness of the improved model.

Reviewer #2:

*The manuscript addresses a relevant and timely topic by investigating the combined effects of CLDHs and recycled aggregate on the performance of RAC and the experimental program is reasonably comprehensive. However, significant revisions are required before publication.Overall, the study has solid foundations and potential, but improvements in novelty positioning, mechanistic justification, statistical rigor, and model integration are necessary.*

The novelty is not clearly positioned relative to existing studies on CLDH-modified cement systems, RAC durability enhancement and TOPSIS-based optimization. The claimed synergistic mechanism between CLDHs and RA appears largely inferred from macroscopic trends rather than mechanistically demonstrated. The authors should clarify what new insight is provided and moderate claims where direct evidence is lacking.

*Response:Thank you for your valuable feedback. We have thoroughly revised the section on innovative contributions in the Discussion portion of the manuscript.****(See the revised draft lines 836-856)***

In summary, this study preliminarily clarifies the synergistic mechanism between CLDHs and RA in enhancing the resistance of RAC to chloride ion penetration. By integrating microscopic analyses and macroscopic performance evaluation, it reveals the pore structure optimization characteristics imparted by CLDHs on RA. An improved TOPSIS evaluation model combining subjective and objective dimensions is established, enabling a comprehensive multi - criteria assessment of the synergistic effect of CLDHs and RA on improving the chloride ion penetration resistance of RAC. This work advances the mix proportion optimization methodology for such concrete and provides adjustable decision - making references for mix proportion selection under varying corrosion environments. The microscopic analysis demonstrates that the optimization of hydration products and refinement of pore structure are critical factors underpinning the macroscopic performance enhancement. It should be noted that this study employs only the DTL-9T rapid chloride permeability test as the macroscopic evaluation indicator for chloride ion penetration resistance. While this method rapidly reflects concrete’s resistance to chloride ion migration, it does not fully capture the entire process of chloride adsorption, binding, and diffusion within concrete. Subsequent research will incorporate metrics such as chloride binding capacity and diffusion coefficients to refine the durability evaluation framework. Furthermore, early - age strength optimization strategies and the mechanism by which CLDHs influence the interfacial transition zone of RA remain key directions for future investigation.

*Furthermore, we have toned down causal assertions that are predicated on "direct evidence" and opted for more cautious formulations such as "suggests that," "is consistent with the hypothesis that," and "indicates a possible synergistic effect."*

The mechanistic interpretation requires stronger quantitative support. XRD analysis is qualitative, SEM observations are descriptive, NMR lacks pore connectivity analysis, and no direct chloride binding measurements are presented. Statistical reliability is also insufficient: no standard deviations, error bars, or significance testing are included despite the multi-factorial design. Incorporating statistical analysis (e.g., ANOVA) is strongly recommended.

*Response:We sincerely appreciate your valuable suggestion. We acknowledge that the analysis in the original draft tends to be predominantly qualitative.This study indirectly characterizes the interconnectivity of pore systems by calculating T2 values, T2 spectrum peak area ratios, and relaxation time distributions. It explicitly acknowledges that while NMR cannot directly measure permeability, it effectively captures variations in pore size distribution. Quantitative analyses in this regard will be further refined in future investigations. Regarding chloride ion binding, the revised manuscript states in Section 5 (Conclusions): "Isothermal adsorption tests for chloride ions were not conducted as part of this study, and such investigations will be prioritized in subsequent research."The study has been supplemented with a saliency analysis.****(See the revised draft lines 277-293，325-334，356-364 and 378-394)***

The significance analysis revealed that both the replacement ratio of recycled aggregates and the dosage of CLDHs exerted a statistically significant influence on the compressive strength of recycled aggregate concrete at all curing ages. At the early age of 3 d, the compressive strength decreased significantly with increasing recycled aggregate content, with the reference group demonstrating markedly superior performance compared to all modified groups. As hydration progressed, the results at 7 and 28 d indicated that an appropriate combination of CLDHs and recycled aggregates could synergistically enhance the compressive strength of concrete [38]. However, excessive dosage of either component or an imbalanced ratio between the two led to a reduction in concrete strength. Among the tested mixtures, RC20-1 (1% CLDHs + 20% recycled aggregates) achieved the highest compressive strength, whereas RC30-6 (6% CLDHs + 30% recycled aggregates) yielded the lowest. At 7 d, the effect of CLDHs dosage became evident, with mixtures containing 1% CLDHs exhibiting superior strength performance. The RC20-1 group significantly outperformed other specimens, while groups with high levels of both recycled aggregates and CLDHs showed a pronounced decline in strength.

At the 28 d core curing age, the groups with 10% recycled aggregate combined with 1% to 6% hydrotalcite, as well as those with 20% recycled aggregate and 1% hydrotalcite, exhibit no significant differences and demonstrate performance levels similar to that of the control group, representing the optimal performance. However, as the substitution rate of recycled aggregate increases to 30% and the hydrotalcite dosage rises to 3% to 6%, a notable decline in compressive strength is observed, with the RC30 - 6 group reaching the lowest value. This indicates that excessively high proportions of recycled aggregate and hydrotalcite exhibit a significant negative synergistic effect, thereby deteriorating the long-term mechanical properties of concrete.

The significance analysis reveals that both the replacement ratio of recycled aggregate and the dosage of calcined hydrotalcite exert a statistically significant influence on the electric flux of RAC. Under the same recycled aggregate replacement ratio, the electric flux of the group with 3% hydrotalcite dosage is significantly lower than that of the groups with 1% and 6% dosages. Specifically, the RC30 - 3 group exhibits the lowest electric flux, indicating superior resistance to chloride ion penetration. Conversely, the electric flux of the 6% hydrotalcite dosage group is markedly higher than that of the control group, demonstrating a significant deterioration in impermeability performance.

At a 3% hydrotalcite dosage, the electric flux exhibited a significant decreasing trend with increasing recycled aggregate replacement ratio. The combined use of high replacement ratios and 3% hydrotalcite substantially enhanced the impermeability.

However, when the CLDHs content reached 6%, the improvement in impermeability across all specimen groups was markedly reduced compared to other experimental sets. This attenuation is attributed to the aggravated "agglomeration effect" of CLDHs at high concentrations, which introduces new structural defects, increases porosity, and induces microcracks, thereby compromising the integrity and continuity of the concrete pore structure and consequently diminishing the resistance to chloride ion migration. At a 6% hydrotalcite dosage, the electric flux increased significantly with rising replacement ratios, indicating a negative synergistic effect. Specimens with 10% recycled aggregate combined with either 1% or 6% hydrotalcite showed no significant difference in electric flux compared to the control group. In contrast, those with 20% or 30% recycled aggregate combined with 1% hydrotalcite exhibited significantly lower electric flux and superior impermeability relative to the control.

The selection of CLDH and RA ranges needs clearer justification, and the limitations of relying solely on ASTM C1202 electrical flux as a durability indicator should be acknowledged. The practical value of the EW–AHP–TOPSIS model would benefit from sensitivity analysis and clearer demonstration of its added value over simple performance ranking.

*Response:Thank you for raising this critical point. This is indeed a highly valuable insight. We acknowledge the limitations of relying solely on ASTM C1202 rapid chloride permeability test results as a durability indicator, and we have elaborated on these constraints in the discussion section. Concurrently, the EW–AHP–TOPSIS model was validated through sensitivity analysis.****(See the revised draft lines 836-856, 674-681 and 717-742)***

In summary, this study preliminarily clarifies the synergistic mechanism between CLDHs and RA in enhancing the resistance of RAC to chloride ion penetration. By integrating microscopic analyses and macroscopic performance evaluation, it reveals the pore structure optimization characteristics imparted by CLDHs on RA. An improved TOPSIS evaluation model combining subjective and objective dimensions is established, enabling a comprehensive multi - criteria assessment of the synergistic effect of CLDHs and RA on improving the chloride ion penetration resistance of RAC. This work advances the mix proportion optimization methodology for such concrete and provides adjustable decision - making references for mix proportion selection under varying corrosion environments. The microscopic analysis demonstrates that the optimization of hydration products and refinement of pore structure are critical factors underpinning the macroscopic performance enhancement. It should be noted that this study employs only the DTL-9T rapid chloride permeability test as the macroscopic evaluation indicator for chloride ion penetration resistance. While this method rapidly reflects concrete’s resistance to chloride ion migration, it does not fully capture the entire process of chloride adsorption, binding, and diffusion within concrete. Subsequent research will incorporate metrics such as chloride binding capacity and diffusion coefficients to refine the durability evaluation framework. Furthermore, early - age strength optimization strategies and the mechanism by which CLDHs influence the interfacial transition zone of RA remain key directions for future investigation.

(5) Model Sensitivity Validation. To further validate the stability of the EW and AHP - enhanced TOPSIS model, a weight sensitivity analysis was conducted to address the limitations of relying solely on entropy weight - based validation. The validation process was designed by integrating the model's characteristics with practical engineering requirements, employing sensitivity analysis. By adjusting the subjective - objective weighting coefficient α (set to 0.3, 0.5, and 0.7, respectively), the relative closeness rankings of alternative solutions under each scenario were computed.

Fig 16 reveals that the results obtained from the EW-TOPSIS method and the EW + AHP - TOPSIS method consistently validate scheme RC30 - 3 (with 3% CLDHs content and 30% RA replacement rate) as exhibiting optimal resistance to chloride ion ingress, while scheme RC30 - 6 (with 6% CLDHs content and 30% RA replacement rate) demonstrates the poorest performance - findings that align with the experimental outcomes. However, notable numerical discrepancies are observed between the two methods. Under the EW + AHP - TOPSIS approach, the closeness degrees for most specimen groups become more polarized. For instance, the closeness of RC30 - 3 rises from 0.76 to 0.79, and that of RC20 - 3 increases from 0.59 to 0.70. Conversely, the closeness of some groups, such as RC0 - 0 and RC10 - 1, diminishes under the EW + AHP - TOPSIS method. This indicates that the adjusted weighting scheme enhances the discriminative capacity of the evaluation results.

To further validate the improved TOPSIS model integrating EW + AHP, sensitivity analysis was conducted to assess the chloride ion penetration resistance of RAC under varying α values. When AHP + EW combination weights assigned β (representing the AHP weighting factor) as 0.5, 0.7, or 0.9 - indicating dominance of AHP or equal influence of both factors - the ranking of RAC specimens remained consistent, specifically: RC30 - 3 > RC20 - 3 > RC30 - 1 > RC20 - 1 > RC10 - 3 > RC10 - 1 > RC10 - 6 > RC0 - 0 > RC20 - 6 > RC30 - 6. Conversely, when α was set to 0.5, 0.7, or 0.9 - reflecting EW dominance or balanced weighting - the rankings showed variability. For example, with α = 0.5, specimen RC10 - 3 ranked fifth, whereas with α = 0.7, it ranked third. These results demonstrate that the rankings produced by the AHP + EW - TOPSIS model remain stable when AHP either predominates or shares equal weight, which markedly exceeds the ranking consistency achievable with the traditional EW - TOPSIS model, thereby confirming the superior robustness of the improved model.

*6. PLOS authors have the option to publish the peer review history of their article (what does this mean?). If published, this will include your full peer review and any attached files.*

*If you choose “no”, your identity will remain anonymous but your review may still be made public.*

*Do you want your identity to be public for this peer review? For information about this choice, including consent withdrawal, please see our Privacy Policy.*

*Reviewer #1: No*

*Reviewer #2: Yes: Zain Ul Abdin*

*Response:We acknowledge the journal's options regarding the disclosure of peer review history and respect the respective choices made by Reviewer 1 and Reviewer 2. We have no objections to these decisions.*

*[NOTE: If reviewer comments were submitted as an attachment file, they will be attached to this email and accessible via the submission site. Please log into your account, locate the manuscript record, and check for the action link "View Attachments". If this link does not appear, there are no attachment files.]*

*To ensure your figures meet our technical requirements, please review our figure guidelines: https://journals.plos.org/plosone/s/figures*

*You may also use PLOS’s free figure tool, NAAS, to help you prepare publication quality figures: https://journals.plos.org/plosone/s/figures#loc-tools-for-figure-preparation.*

*NAAS will assess whether your figures meet our technical requirements by comparing each figure against our figure specifications.*

*Response: Thank you for your valuable comments and for reminding us to comply with the figure requirements of PLOS ONE. We have carefully reviewed the journal’s figure guidelines and used the free NAAS tool to check and optimize all figures in the manuscript. All figures have been converted to TIFF format with LZW compression, set to 300–600 dpi, adjusted to the required dimensions, and standardized in font, color mode, and layout. We have also prepared the original uncropped micrographs and test images as a single supporting information file named S1_raw_images.pdf to ensure data transparency and integrity. All figures now fully meet the publication standards of PLOS ONE.*

PONE-D-26-05199_Technical_Queries_Table

The abstract and results state that the mix with 1% CLDHs and 20% RA yielded the best compressive strength, while 3% CLDHs and 30% RA was optimal for chloride resistance. Please explain how an engineering designer should balance these two objectives (mechanical strength vs. permeability) in practical applications?

*Response:Thank you for raising this critical engineering issue. In practical engineering applications, trade-offs should be made based on service environment and design lifespan. We have added a new subsection titled "4.3 Economic Feasibility Analysis Model" in the revised manuscript under Section 4, "Optimization of RAC Anti-Chloride Ion Erosion Performance Scheme Based on the Improved TOPSIS Method Integrating Entropy Weight and AHP."****(See the revised draft lines 771-783)***

In summary, if ranked solely by a single parameter, RC20 - 1 yields the optimal compressive strength, whereas RC30 - 3 ranks highest when considering only electrical flux. These conflicting outcomes based on distinct performance indicators pose challenges for engineering decision - making. To address this issue, the EW + AHP - TOPSIS model proposed in this study integrates six criteria - including compressive strength, electrical flux, and multi - scale pore structure characteristics - by combining subjective and objective weighting methods. The approach further incorporates economic considerations to provide a flexible decision - support framework for selecting mix proportions under varying corrosion environments, as illustrated in Table 8.

**Table 8. Recommended decision alternatives**

| Engineering context | Priority metrics | Recommended formulation ratio |
| --- | --- | --- |
| Non-corrosive environment | High intensity prioritization | RC20-1 |
| Moderate corrosive environment | Overall balance | RC20-3 |
| Highly corrosive environment | Priority of impermeability resistance | RC30-3 |

Considering the use of Recycled Aggregates (RA) and Calcined Layered Double Hydroxides (CLDHs), please provide a brief analysis of the economic feasibility of this approach. Does the cost associated with the calcination process of hydrotalcite at 500°C outweigh the long-term technical benefits, such as enhanced concrete durability?

*Response:We appreciate your attention to the economic aspect of the project. In the revised manuscript, a new subsection titled "4.3. Economic Feasibility Analysis Model" has been added under Section 4, “Optimization of RAC Chloride Ion Erosion Resistance Solutions Based on Entropy Weight and AHP-Enhanced TOPSIS Method.”****(See the revised draft lines 744-770)***

4.3. Economic feasibility analysis model

Based on the optimized chloride ion corrosion resistance performance scheme, this study evaluates the cost-effectiveness of RAC and provides data support for selecting optimal construction materials and building strategies. The assessment primarily encompasses upstream processes including raw material acquisition, material transportation, and concrete preparation. The total cost per cubic meter of concrete is calculated using Eq (16), with computational outcomes presented in Table 5. For each material category, comprehensive cost components are considered, covering initial procurement price, transportation expenses, energy consumption, equipment depreciation, and yield loss costs [49].

| $C=\sum_{i=1}^{7} C_{i}$ | （16） |
| --- | --- |

Where *C_i_* denotes the cost of material *i*.

**Table 7. Economic assessment of the radial artery craft**

| Specimen NO. | Cost/USD·m^-3^ | | | | | | | Cost/USD·m^-3^ |
| --- | --- | --- | --- | --- | --- | --- | --- | --- |
|  | OPC | CLDHs | Water | River sand | Crushed  stone | Recycled aggregate | water reduce |  |
| RC0-0 | 30.6 | 0 | 0.0 | 1.1 | 16.5 | 0 | 41.3 | 89.5 |
| RC10-1 | 30.3 | 9.3 | 0.0 | 1.1 | 14.8 | 0.3 | 41.3 | 97.1 |
| RC10-3 | 29.6 | 27.5 | 0.0 | 1.1 | 14.8 | 0.3 | 41.3 | 114.6 |
| RC10-6 | 28.7 | 55.0 | 0.0 | 1.1 | 14.8 | 0.3 | 41.3 | 141.2 |
| RC20-1 | 30.3 | 9.2 | 0.0 | 1.1 | 13.2 | 0.6 | 41.3 | 95.7 |
| RC20-3 | 29.6 | 27.5 | 0.0 | 1.1 | 13.2 | 0.6 | 41.3 | 113.3 |
| RC20-6 | 28.7 | 55.0 | 0.0 | 1.1 | 13.2 | 0.6 | 41.3 | 139.9 |
| RC30-1 | 30.3 | 9.2 | 0.0 | 1.1 | 11.5 | 0.9 | 41.3 | 94.3 |
| RC30-3 | 29.6 | 27.5 | 0.0 | 1.1 | 11.5 | 0.9 | 41.3 | 111.9 |
| RC30-6 | 28.7 | 55.0 | 0.0 | 1.1 | 11.5 | 0.9 | 41.3 | 138.5 |

Table 7 indicates that CLDHs contribute dominantly to cost escalation, while RA alleviates the cost burden. Compared to RC20 - 1, which achieved the optimal compressive strength, RC30 - 3 - exhibiting the best chloride ion penetration resistance - incurred an additional cost of 16.2 USD/m³, representing an increase of approximately 17%. Although CLDHs carry a relatively high unit price, their incorporation rate ranges only from 1% to 6%, resulting in a limited cost increment per cubic meter of concrete. More importantly, CLDHs significantly enhance the chloride erosion resistance of RAC, extend the service life of structures in saline soil environments, and reduce lifecycle maintenance costs. For moderate to severe corrosive environments such as coastal and saline - alkali regions, this cost increment is acceptable, thereby demonstrating practical engineering applicability. However, CLDHs - modified concrete is not recommended for non - corrosive environments or temporary structures.

In section 2.3.1, it is mentioned that the average of three cubic specimens was used for each compressive strength group. Was a similar repetition procedure followed for other tests, such as chloride ion penetration and NMR? Specifying the exact number of tested specimens for each section would enhance the paper's scientific rigor.

*Response:Thank you for emphasizing the importance of experimental rigor. In Section 2.3 (Test Method) of the revised manuscript, we will explicitly specify the sample sizes for each test in a tabular format.****(See the revised draft lines 186-192)***

This study conducted compressive strength tests, chloride ion permeability tests, X-ray diffraction (XRD) analyses, scanning electron microscopy (SEM) observations, and nuclear magnetic resonance (NMR) measurements. The actual sample sizes for each experimental procedure are presented in Table 4.

**Table 4. Actual sample sizes at each experimental phase**

| Test item | Specimen specification/mm | Number of specimens per mix proportion group/pcs. | times of repetition/times |
| --- | --- | --- | --- |
| Compressive strength test （3d, 7d, 28d） | 100×100 | 3 | 3 |
| Chloride ion (Cl^-^) penetration test（28d） | Φ100 ×50 | 3 | 3 |
| XRD test | Fine powder form | 1 | 1 |
| SEM test | Block | 2 | 2 |
| NMR test | 40×40 | 3 | 3 |

In the 3-day compressive strength results, it was observed that the strength of all groups was lower than the control group. The author attributes this to water absorption by CLDHs and defects in the Interface Transition Zone (ITZ). Does the author suggest any strategy to improve early-age strength in this type of concrete, such as the use of accelerating admixtures?

*Response:Thank you for your constructive suggestions. We have incorporated a discussion on the limitations of 3 d compressive strength in the revised manuscript. The paper does not propose optimization strategies for enhancing the early-age strength of this type of concrete, which will be addressed in future research.****(See the revised draft lines 836-856)***

In summary, this study preliminarily clarifies the synergistic mechanism between CLDHs and RA in enhancing the resistance of RAC to chloride ion penetration. By integrating microscopic analyses and macroscopic performance evaluation, it reveals the pore structure optimization characteristics imparted by CLDHs on RA. An improved TOPSIS evaluation model combining subjective and objective dimensions is established, enabling a comprehensive multi - criteria assessment of the synergistic effect of CLDHs and RA on improving the chloride ion penetration resistance of RAC. This work advances the mix proportion optimization methodology for such concrete and provides adjustable decision - making references for mix proportion selection under varying corrosion environments. The microscopic analysis demonstrates that the optimization of hydration products and refinement of pore structure are critical factors underpinning the macroscopic performance enhancement. It should be noted that this study employs only the DTL-9T rapid chloride permeability test as the macroscopic evaluation indicator for chloride ion penetration resistance. While this method rapidly reflects concrete’s resistance to chloride ion migration, it does not fully capture the entire process of chloride adsorption, binding, and diffusion within concrete. Subsequent research will incorporate metrics such as chloride binding capacity and diffusion coefficients to refine the durability evaluation framework. Furthermore, early - age strength optimization strategies and the mechanism by which CLDHs influence the interfacial transition zone of RA remain key directions for future investigation.

The author utilized an improved TOPSIS model integrating Entropy Weight (EW) and Analytic Hierarchy Process (AHP). Please clarify how many expert opinions were considered for the subjective weighting (AHP) or what standards were followed to ensure the model's reliability across different engineering environments?

*Response:Thank you for your attention to the methodological rigor. We have supplemented the content in Section 4.2, "Improved TOPSIS Model with Entropy Weight and AHP," of the revised manuscript.****(See the revised draft lines 652-662, 674-681 and 717-742)***

(1)AHP Weight Determination. In this study, the subjective weights for AHP were determined through consultations with five domain experts, comprising two specialists in building materials and concrete durability, one in structural engineering and life - cycle design, one in engineering economics and project management, and one in engineering practices specific to saline soil regions. Using the 1 - 9 scale method, pairwise comparisons were conducted for the evaluation indicators - compressive strength, electrical flux, non - harmful porosity, slightly harmful porosity, harmful porosity, and highly harmful porosity - to construct a comparison matrix M. Subsequently, the eigenvector method was employed to determine the weights, followed by a consistency check, resulting in the weight matrix W_2_ = w_2j_, as presented in Eq. (13).

(5) Model Sensitivity Validation. To further validate the stability of the EW and AHP - enhanced TOPSIS model, a weight sensitivity analysis was conducted to address the limitations of relying solely on entropy weight - based validation. The validation process was designed by integrating the model's characteristics with practical engineering requirements, employing sensitivity analysis. By adjusting the subjective - objective weighting coefficient α (set to 0.3, 0.5, and 0.7, respectively), the relative closeness rankings of alternative solutions under each scenario were computed.

Fig 16 reveals that the results obtained from the EW-TOPSIS method and the EW + AHP - TOPSIS method consistently validate scheme RC30 - 3 (with 3% CLDHs content and 30% RA replacement rate) as exhibiting optimal resistance to chloride ion ingress, while scheme RC30 - 6 (with 6% CLDHs content and 30% RA replacement rate) demonstrates the poorest performance - findings that align with the experimental outcomes. However, notable numerical discrepancies are observed between the two methods. Under the EW + AHP - TOPSIS approach, the closeness degrees for most specimen groups become more polarized. For instance, the closeness of RC30 - 3 rises from 0.76 to 0.79, and that of RC20 - 3 increases from 0.59 to 0.70. Conversely, the closeness of some groups, such as RC0 - 0 and RC10 - 1, diminishes under the EW + AHP - TOPSIS method. This indicates that the adjusted weighting scheme enhances the discriminative capacity of the evaluation results.

To further validate the improved TOPSIS model integrating EW + AHP, sensitivity analysis was conducted to assess the chloride ion penetration resistance of RAC under varying α values. When AHP + EW combination weights assigned β (representing the AHP weighting factor) as 0.5, 0.7, or 0.9 - indicating dominance of AHP or equal influence of both factors - the ranking of RAC specimens remained consistent, specifically: RC30 - 3 > RC20 - 3 > RC30 - 1 > RC20 - 1 > RC10 - 3 > RC10 - 1 > RC10 - 6 > RC0 - 0 > RC20 - 6 > RC30 - 6. Conversely, when α was set to 0.5, 0.7, or 0.9 - reflecting EW dominance or balanced weighting - the rankings showed variability. For example, with α = 0.5, specimen RC10 - 3 ranked fifth, whereas with α = 0.7, it ranked third. These results demonstrate that the rankings produced by the AHP + EW - TOPSIS model remain stable when AHP either predominates or shares equal weight, which markedly exceeds the ranking consistency achievable with the traditional EW - TOPSIS model, thereby confirming the superior robustness of the improved model.
