# Supplementary material for: Study on chloride ion erosion resistance of recycled aggregate concrete based on an improved TOPSIS model integrating entropy weight and AHP
Source: PLoS One. 2026 Jul 22;21(7):e0352439. doi: 10.1371/journal.pone.0352439 (PMC13390877; doi:10.1371/journal.pone.0352439)
Supplement: Peer review history

# Peer review: Decision Letter 0

**DOI:** 10.1371/journal.pone.0352439.r001

**Authors:** Dola Sundeep

28 Mar 2026

PONE-D-26-05199

Study on Chloride Ion Erosion Resistance of Recycled Aggregate Concrete Based on an Improved TOPSIS Model Integrating Entropy Weight and AHP

PLOS One

Dear Dr. Zheng,

Thank you for submitting your manuscript to PLOS ONE. After careful consideration, we feel that it has merit but does not fully meet PLOS ONE’s publication criteria as it currently stands. Therefore, we invite you to submit a revised version of the manuscript that addresses the points raised during the review process.

Please submit your revised manuscript by May 12 2026 11:59PM. If you will need more time than this to complete your revisions, please reply to this message or contact the journal office at plosone@plos.org. When you're ready to submit your revision, log on to https://www.editorialmanager.com/pone/ and select the 'Submissions Needing Revision' folder to locate your manuscript file.

Please include the following items when submitting your revised manuscript:

- A letter that responds to each point raised by the academic editor and reviewer(s). You should upload this letter as a separate file labeled 'Response to Reviewers'.
- A marked-up copy of your manuscript that highlights changes made to the original version. You should upload this as a separate file labeled 'Revised Manuscript with Track Changes'.
- An unmarked version of your revised paper without tracked changes. You should upload this as a separate file labeled 'Manuscript'.

If you would like to make changes to your financial disclosure, please include your updated statement in your cover letter. Guidelines for resubmitting your figure files are available below the reviewer comments at the end of this letter.

If applicable, we recommend that you deposit your laboratory protocols in protocols.io to enhance the reproducibility of your results. Protocols.io assigns your protocol its own identifier (DOI) so that it can be cited independently in the future. For instructions see: https://journals.plos.org/plosone/s/submission-guidelines#loc-laboratory-protocols. Additionally, PLOS ONE offers an option for publishing peer-reviewed Lab Protocol articles, which describe protocols hosted on protocols.io. Read more information on sharing protocols at https://plos.org/protocols?utm_medium=editorial-email&utm_source=authorletters&utm_campaign=protocols.

As the corresponding author, your ORCID iD is verified in the submission system and will appear in the published article. PLOS supports the use of ORCID, and we encourage all coauthors to register for an ORCID iD and use it as well. Please encourage your coauthors to verify their ORCID iD within the submission system before final acceptance, as unverified ORCID iDs will not appear in the published article. *Only*  the individual author can complete the verification step; PLOS staff *cannot*  verify ORCID iDs on behalf of authors.

We look forward to receiving your revised manuscript.

Kind regards,

Dola Sundeep

Academic Editor

PLOS One

Journal Requirements:

When submitting your revision, we need you to address these additional requirements.

1. Please ensure that your manuscript meets PLOS ONE's style requirements, including those for file naming. The PLOS ONE style templates can be found at

https://journals.plos.org/plosone/s/file?id=wjVg/PLOSOne_formatting_sample_main_body.pdf and

https://journals.plos.org/plosone/s/file?id=ba62/PLOSOne_formatting_sample_title_authors_affiliations.pdf

2. We note that this submission includes NMR spectroscopy data. We would recommend that you include the following information in your methods section or as Supporting Information files:

1) The make/source of the NMR instrument used in your study, as well as the magnetic field strength. For each individual experiment, please also list: the nucleus being measured; the sample concentration; the solvent in which the sample is dissolved and if solvent signal suppression was used; the reference standard and the temperature.

2) A list of the chemical shifts for all compounds characterised by NMR spectroscopy, specifying, where relevant: the chemical shift (δ), the multiplicity and the coupling constants (in Hz), for the appropriate nuclei used for assignment.

3)The full integrated NMR spectrum, clearly labelled with the compound name and chemical structure.

We also strongly encourage authors to provide primary NMR data files, in particular for new compounds which have not been characterised in the existing literature. Authors should provide the acquisition data, FID files and processing parameters for each experiment, clearly labelled with the compound name and identifier, as well as a structure file for each provided dataset. See our list of recommended repositories here: https://journals.plos.org/plosone/s/recommended-repositories

3. PLOS requires an ORCID iD for the corresponding author in Editorial Manager on papers submitted after December 6th, 2016. Please ensure that you have an ORCID iD and that it is validated in Editorial Manager. To do this, go to ‘Update my Information’ (in the upper left-hand corner of the main menu), and click on the Fetch/Validate link next to the ORCID field. This will take you to the ORCID site and allow you to create a new iD or authenticate a pre-existing iD in Editorial Manager.

4. We note that the grant information you provided in the ‘Funding Information’ and ‘Financial Disclosure’ sections do not match.

When you resubmit, please ensure that you provide the correct grant numbers for the awards you received for your study in the ‘Funding Information’ section.

5. Thank you for stating the following financial disclosure:

The author(s) received no specific funding for this work.

At this time, please address the following queries:

a) Please clarify the sources of funding (financial or material support) for your study. List the grants or organizations that supported your study, including funding received from your institution.

b) State what role the funders took in the study. If the funders had no role in your study, please state: “The funders had no role in study design, data collection and analysis, decision to publish, or preparation of the manuscript.”

c) If any authors received a salary from any of your funders, please state which authors and which funders.

d) If you did not receive any funding for this study, please state: “The authors received no specific funding for this work.”

Please include your amended statements within your cover letter; we will change the online submission form on your behalf.

6. Thank you for stating the following in the Acknowledgments Section of your manuscript:

This research was funded by the Xinjiang Uygur Autonomous Region Natural Science Foundation (No. 2025D01A05), the China College Students’ Innovation and Entrepreneurship Development Program (No. 202510763009), and the Xinjiang UygurAutonomous Region College Students’ Innovation and Entrepreneurship Training Program (No. S202510763042).

We note that you have provided funding information that is not currently declared in your Funding Statement. However, funding information should not appear in the Acknowledgments section or other areas of your manuscript. We will only publish funding information present in the Funding Statement section of the online submission form.

Please remove any funding-related text from the manuscript and let us know how you would like to update your Funding Statement. Currently, your Funding Statement reads as follows:

The author(s) received no specific funding for this work.

Please include your amended statements within your cover letter; we will change the online submission form on your behalf.

7. We note that your Data Availability Statement is currently as follows: All relevant data are within the manuscript and its Supporting Information files.

Please confirm at this time whether or not your submission contains all raw data required to replicate the results of your study. Authors must share the “minimal data set” for their submission. PLOS defines the minimal data set to consist of the data required to replicate all study findings reported in the article, as well as related metadata and methods (https://journals.plos.org/plosone/s/data-availability#loc-minimal-data-set-definition).

For example, authors should submit the following data:

- The values behind the means, standard deviations and other measures reported;

- The values used to build graphs;

- The points extracted from images for analysis.

Authors do not need to submit their entire data set if only a portion of the data was used in the reported study.

If your submission does not contain these data, please either upload them as Supporting Information files or deposit them to a stable, public repository and provide us with the relevant URLs, DOIs, or accession numbers. For a list of recommended repositories, please see https://journals.plos.org/plosone/s/recommended-repositories.

If there are ethical or legal restrictions on sharing a de-identified data set, please explain them in detail (e.g., data contain potentially sensitive information, data are owned by a third-party organization, etc.) and who has imposed them (e.g., an ethics committee). Please also provide contact information for a data access committee, ethics committee, or other institutional body to which data requests may be sent. If data are owned by a third party, please indicate how others may request data access.

8. Please amend your list of authors on the manuscript to ensure that each author is linked to an affiliation. Authors’ affiliations should reflect the institution where the work was done (if authors moved subsequently, you can also list the new affiliation stating “current affiliation:….” as necessary).

9. Thank you for stating the following in the Competing Interests/Financial Disclosure section:

The authors have declared that no competing interests exist.

We note that one or more of the authors are employed by a commercial company: Kashi Zhengxin Construction Engineering Testing Co., Ltd

a. Please provide an amended Funding Statement declaring this commercial affiliation, as well as a statement regarding the Role of Funders in your study. If the funding organization did not play a role in the study design, data collection and analysis, decision to publish, or preparation of the manuscript and only provided financial support in the form of authors' salaries and/or research materials, please review your statements relating to the author contributions, and ensure you have specifically and accurately indicated the role(s) that these authors had in your study. You can update author roles in the Author Contributions section of the online submission form.

Please also include the following statement within your amended Funding Statement.

“The funder provided support in the form of salaries for authors [insert relevant initials], but did not have any additional role in the study design, data collection and analysis, decision to publish, or preparation of the manuscript. The specific roles of these authors are articulated in the ‘author contributions’ section.”

If your commercial affiliation did play a role in your study, please state and explain this role within your updated Funding Statement.

b. Please also provide an updated Competing Interests Statement declaring this commercial affiliation along with any other relevant declarations relating to employment, consultancy, patents, products in development, or marketed products, etc.

Within your Competing Interests Statement, please confirm that this commercial affiliation does not alter your adherence to all PLOS ONE policies on sharing data and materials by including the following statement: "This does not alter our adherence to PLOS ONE policies on sharing data and materials.” (as detailed online in our guide for authors http://journals.plos.org/plosone/s/competing-interests) . If this adherence statement is not accurate and there are restrictions on sharing of data and/or materials, please state these. Please note that we cannot proceed with consideration of your article until this information has been declared.

Please include both an updated Funding Statement and Competing Interests Statement in your cover letter. We will change the online submission form on your behalf.

10. If the reviewer comments include a recommendation to cite specific previously published works, please review and evaluate these publications to determine whether they are relevant and should be cited. There is no requirement to cite these works unless the editor has indicated otherwise.

Additional Editor Comments:

Dear Xiumei Zheng

We have now received the required number of reviewer reports for your manuscript titled:

**“Study on Chloride Ion Erosion Resistance of Recycled Aggregate Concrete Based on an Improved TOPSIS Model Integrating Entropy Weight and AHP”** (Manuscript Number: PONE-D-26-05199), submitted to *PLOS ONE*.

Based on the reviewers’ comments, the Editorial Board has reached a decision of **Major Revision**.

Please carefully address all reviewer comments and submit a revised version of your manuscript within the specified timeframe.

Thank you for your interest in and contribution to *PLOS ONE*.

Kind regards,

Dr. Dola Sundeep

Academic Editor

*PLOS ONE*

[Note: HTML markup is below. Please do not edit.]

Reviewers' comments:

Reviewer's Responses to Questions

**Comments to the Author**

1. Is the manuscript technically sound, and do the data support the conclusions?

The manuscript must describe a technically sound piece of scientific research with data that supports the conclusions. Experiments must have been conducted rigorously, with appropriate controls, replication, and sample sizes. The conclusions must be drawn appropriately based on the data presented.

Reviewer #1: Yes

Reviewer #2: Yes

**********

2. Has the statistical analysis been performed appropriately and rigorously?

Reviewer #1: Yes

Reviewer #2: Yes

**********

3. Have the authors made all data underlying the findings in their manuscript fully available?

The PLOS Data policy requires authors to make all data underlying the findings described in their manuscript fully available without restriction, with rare exception (please refer to the Data Availability Statement in the manuscript PDF file). The data should be provided as part of the manuscript or its supporting information, or deposited to a public repository. For example, in addition to summary statistics, the data points behind means, medians and variance measures should be available. If there are restrictions on publicly sharing data—e.g. participant privacy or use of data from a third party—those must be specified.

Reviewer #1: Yes

Reviewer #2: Yes

**********

4. Is the manuscript presented in an intelligible fashion and written in standard English?

PLOS ONE does not copyedit accepted manuscripts, so the language in submitted articles must be clear, correct, and unambiguous. Any typographical or grammatical errors should be corrected at revision, so please note any specific errors here.

Reviewer #1: Yes

Reviewer #2: Yes

**********

5. Review Comments to the Author

Please use the space provided to explain your answers to the questions above. You may also include additional comments for the author, including concerns about dual publication, research ethics, or publication ethics. (Please upload your review as an attachment if it exceeds 20,000 characters)

Reviewer #1: The manuscript is scientifically well-constructed. If the author provides a convincing explanation regarding the economic feasibility (as requested in the comments) and clarifies the weighting model, the paper will be highly valuable for the field. I recommend Minor Revision leading toward acceptance.

Reviewer #2: The manuscript addresses a relevant and timely topic by investigating the combined effects of CLDHs and recycled aggregate on the performance of RAC and the experimental program is reasonably comprehensive. However, significant revisions are required before publication.

The novelty is not clearly positioned relative to existing studies on CLDH-modified cement systems, RAC durability enhancement and TOPSIS-based optimization. The claimed synergistic mechanism between CLDHs and RA appears largely inferred from macroscopic trends rather than mechanistically demonstrated. The authors should clarify what new insight is provided and moderate claims where direct evidence is lacking.

The mechanistic interpretation requires stronger quantitative support. XRD analysis is qualitative, SEM observations are descriptive, NMR lacks pore connectivity analysis, and no direct chloride binding measurements are presented. Statistical reliability is also insufficient: no standard deviations, error bars, or significance testing are included despite the multi-factorial design. Incorporating statistical analysis (e.g., ANOVA) is strongly recommended.

The selection of CLDH and RA ranges needs clearer justification, and the limitations of relying solely on ASTM C1202 electrical flux as a durability indicator should be acknowledged. The practical value of the EW–AHP–TOPSIS model would benefit from sensitivity analysis and clearer demonstration of its added value over simple performance ranking.

Overall, the study has solid foundations and potential, but improvements in novelty positioning, mechanistic justification, statistical rigor, and model integration are necessary.

**********

6. PLOS authors have the option to publish the peer review history of their article (what does this mean?). If published, this will include your full peer review and any attached files.

If you choose “no”, your identity will remain anonymous but your review may still be made public.

**Do you want your identity to be public for this peer review?**  For information about this choice, including consent withdrawal, please see our Privacy Policy.

Reviewer #1: No

Reviewer #2: **Yes:** Zain Ul Abdin

**********

[NOTE: If reviewer comments were submitted as an attachment file, they will be attached to this email and accessible via the submission site. Please log into your account, locate the manuscript record, and check for the action link "View Attachments". If this link does not appear, there are no attachment files.]

To ensure your figures meet our technical requirements, please review our figure guidelines: https://journals.plos.org/plosone/s/figures

You may also use PLOS’s free figure tool, NAAS, to help you prepare publication quality figures: https://journals.plos.org/plosone/s/figures#loc-tools-for-figure-preparation.

NAAS will assess whether your figures meet our technical requirements by comparing each figure against our figure specifications.

Attachment Submitted filename: PONE-D-26-05199_Annotated_Manuscript.pdf.pdf

Attachment Submitted filename: PONE-D-26-05199_Technical_Queries_Table.pdf

Attachment Submitted filename: PONE-D-26-05199_Cover_Letter_to_Editor.pdf

# Author comment: Author response to Decision Letter 1

**DOI:** 10.1371/journal.pone.0352439.r002

27 Apr 2026

The author team would like to express our sincere gratitude to the editor and all reviewers for their comprehensive, in-depth, and constructive comments on our manuscript titled “Study on chloride ion erosion resistance of recycled aggregate concrete based on an improved TOPSIS model integrating entropy weight and AHP” (Manuscript Number: PONE-D-26-05199). These comments have played a crucial role in improving the quality of our manuscript and guiding our subsequent research, and we have carefully addressed each comment and completed corresponding revisions. All revised sections in the manuscript are marked in red for the convenience of the editor and reviewers, and we have also conducted a comprehensive review of the entire manuscript to ensure its academic rigor, logical coherence, and overall integrity.

In response to the journal’s additional requirements, we have completed the following revisions: First, we have comprehensively reviewed and revised the manuscript in strict accordance with PLOS ONE’s style requirements, including standardized file naming, to ensure full compliance with the journal’s submission standards. Second, regarding the NMR spectroscopy data involved in the study, we have clarified that NMR was only used to characterize the pore structure of concrete materials (not for compound identification), and supplemented detailed information about the NMR instrument model, manufacturer, testing conditions, and key experimental parameters in the methodology section, along with clearly annotated spectra and corresponding analyses. Third, we have completed the registration and validation of the corresponding author’s ORCID iD in the Editorial Manager system, ensuring the information is accurate and valid. Fourth, we have thoroughly reviewed and aligned the funding information in both the “Funding” and “Financial Disclosure” sections to ensure consistency, correctly entered the accurate project number, and updated the financial disclosure statement as required. Fifth, we have removed all funding-related content from the main text and acknowledgments section of the manuscript, and updated the funding statement to clearly list the supporting funds and the role of the funders. Sixth, we have confirmed that the submitted manuscript contains all the minimal data sets necessary for replicating the research results, including original data, chart values, image analysis data, and related metadata, and improved the data availability statement in accordance with the journal’s requirements. Seventh, we have verified and revised the author list, ensuring each author is accurately linked to their institutional affiliation at the time of the research, and annotated current affiliations where applicable. Eighth, we have declared the commercial affiliation of author Li Chuikan (employed by Kashi Zhengxin Construction Engineering Testing Co., Ltd.) in the cover letter and Competing Interests Statement, clarified the company’s role in the study, elaborated on the author’s contributions, and confirmed that this commercial affiliation does not affect adherence to PLOS ONE’s data and material sharing policies. Ninth, we have verified that the current review comments do not require adding specific references, so no supplementary amendments to the reference section are needed. Tenth, we have carefully reviewed the journal’s figure guidelines, used the free NAAS tool to check and optimize all figures, converted them to TIFF format with LZW compression (300–600 dpi), adjusted them to the required dimensions, standardized fonts, color modes, and layouts, and prepared the original uncropped micrographs and test images as a supporting information file named S1_raw_images.pdf to ensure data transparency.

In response to Reviewer #1’s comments: We have added a new section on the economic feasibility analysis of CLDHs-modified recycled aggregate concrete, evaluating the cost-effectiveness from the aspects of raw material acquisition, transportation, and concrete preparation, calculating the total cost per cubic meter of concrete, and analyzing the cost impact of CLDHs and RA, confirming the practical engineering applicability of the scheme in moderate to severe corrosive environments. Additionally, we have supplemented details on the AHP weight determination (consulting 5 domain experts to construct a comparison matrix and conduct consistency checks) and model sensitivity validation (adjusting the subjective-objective weighting coefficient α to verify the stability and discriminative capacity of the improved EW + AHP-TOPSIS model), clarifying the weighting model and its reliability.

In response to Reviewer #2’s comments: We have revised the discussion section to clarify the research novelty, specifying the new insights provided by this study (preliminarily clarifying the synergistic mechanism between CLDHs and RA, establishing an improved TOPSIS model, and providing a decision-support framework for mix proportion selection) and toned down overly causal assertions to ensure rigor. We have supplemented statistical significance analysis (ANOVA) for compressive strength and electric flux, clarifying the significant effects of RA replacement ratio and CLDHs dosage on concrete performance. We have acknowledged the limitations of relying solely on ASTM C1202 electrical flux as a durability indicator and elaborated on future research directions (incorporating chloride binding capacity and diffusion coefficients). We have also supplemented the justification for the selection range of CLDHs and RA, and completed the sensitivity analysis of the EW–AHP–TOPSIS model to demonstrate its added value over simple performance ranking.

In response to the technical queries: We have added a decision-support framework based on the improved TOPSIS model, providing recommended mix proportions for different engineering environments (non-corrosive, moderate corrosive, highly corrosive) to balance mechanical strength and permeability. We have supplemented the economic feasibility analysis as mentioned above, clarifying that the limited cost increment of CLDHs is acceptable in corrosive environments due to long-term durability benefits. We have explicitly specified the sample sizes for each test (compressive strength, chloride ion penetration, XRD, SEM, NMR) in a tabular format to enhance scientific rigor. We have acknowledged the limitation of low early-age (3d) compressive strength and indicated that early-age strength optimization strategies will be a key direction for future research. We have also supplemented the basis for AHP subjective weighting (5 domain experts) and model sensitivity validation results to ensure the model’s reliability across different engineering environments.

Furthermore, we respect the choices of Reviewer #1 (anonymous) and Reviewer #2 (public identity: Zain Ul Abdin) regarding the disclosure of peer review history and have no objections. We have made every effort to address all comments and requirements thoroughly, and we earnestly hope that the revised manuscript meets the publication criteria of PLOS ONE and contributes to the academic value of the journal. Once again, we extend our sincere appreciation to the editor and reviewers for their professional support and guidance.

Attachment Submitted filename: Responses to Reviewer s Comments PONE-D-26-05199.doc

# Peer review: Decision Letter 1

**DOI:** 10.1371/journal.pone.0352439.r003

**Authors:** Gaurav Arora

10 Jun 2026

Study on chloride ion erosion resistance of recycled aggregate concrete based on an improved TOPSIS model integrating entropy weight and AHP

PONE-D-26-05199R1

Dear Dr. Zheng,

We’re pleased to inform you that your manuscript has been judged scientifically suitable for publication and will be formally accepted for publication once it meets all outstanding technical requirements.

Within one week, you’ll receive an e-mail detailing the required amendments. When these have been addressed, you’ll receive a formal acceptance letter and your manuscript will be scheduled for publication.

An invoice will be generated when your article is formally accepted. Please note, if your institution has a publishing partnership with PLOS and your article meets the relevant criteria, all or part of your publication costs will be covered. Please make sure your user information is up-to-date by logging into Editorial Manager at Editorial Manager® and clicking the ‘Update My Information' link at the top of the page. For questions related to billing, please contact billing support.

If your institution or institutions have a press office, please notify them about your upcoming paper to help maximize its impact. If they’ll be preparing press materials, please inform our press team as soon as possible -- no later than 48 hours after receiving the formal acceptance. Your manuscript will remain under strict press embargo until 2 pm Eastern Time on the date of publication. For more information, please contact onepress@plos.org.

Kind regards,

Gaurav Arora, Ph.D

Academic Editor

PLOS One

Additional Editor Comments (optional):

The reviewers have made a decision on the manuscript.

Reviewers' comments:

Reviewer's Responses to Questions

**Comments to the Author**

1. If the authors have adequately addressed your comments raised in a previous round of review and you feel that this manuscript is now acceptable for publication, you may indicate that here to bypass the “Comments to the Author” section, enter your conflict of interest statement in the “Confidential to Editor” section, and submit your "Accept" recommendation.

Reviewer #1: (No Response)

Reviewer #2: All comments have been addressed

**********

2. Is the manuscript technically sound, and do the data support the conclusions?

The manuscript must describe a technically sound piece of scientific research with data that supports the conclusions. Experiments must have been conducted rigorously, with appropriate controls, replication, and sample sizes. The conclusions must be drawn appropriately based on the data presented.

Reviewer #1: (No Response)

Reviewer #2: Yes

**********

3. Has the statistical analysis been performed appropriately and rigorously?

Reviewer #1: (No Response)

Reviewer #2: Yes

**********

4. Have the authors made all data underlying the findings in their manuscript fully available?

The PLOS Data policy requires authors to make all data underlying the findings described in their manuscript fully available without restriction, with rare exception (please refer to the Data Availability Statement in the manuscript PDF file). The data should be provided as part of the manuscript or its supporting information, or deposited to a public repository. For example, in addition to summary statistics, the data points behind means, medians and variance measures should be available. If there are restrictions on publicly sharing data—e.g. participant privacy or use of data from a third party—those must be specified.

Reviewer #1: (No Response)

Reviewer #2: Yes

**********

5. Is the manuscript presented in an intelligible fashion and written in standard English?

PLOS ONE does not copyedit accepted manuscripts, so the language in submitted articles must be clear, correct, and unambiguous. Any typographical or grammatical errors should be corrected at revision, so please note any specific errors here.

Reviewer #1: (No Response)

Reviewer #2: Yes

**********

6. Review Comments to the Author

Please use the space provided to explain your answers to the questions above. You may also include additional comments for the author, including concerns about dual publication, research ethics, or publication ethics. (Please upload your review as an attachment if it exceeds 20,000 characters)

Reviewer #1: (No Response)

Reviewer #2: (No Response)

**********

7. PLOS authors have the option to publish the peer review history of their article (what does this mean?). If published, this will include your full peer review and any attached files.

If you choose “no”, your identity will remain anonymous but your review may still be made public.

**Do you want your identity to be public for this peer review?**  For information about this choice, including consent withdrawal, please see our Privacy Policy.

Reviewer #1: No

Reviewer #2: No

**********

# Editor report: Acceptance letter

**DOI:** 10.1371/journal.pone.0352439.r004

**Authors:** Gaurav Arora

PONE-D-26-05199R1

PLOS One

Dear Dr. Zheng,

I'm pleased to inform you that your manuscript has been deemed suitable for publication in PLOS One. Congratulations! Your manuscript is now being handed over to our production team.

At this stage, our production department will prepare your paper for publication. This includes ensuring the following:

* All references, tables, and figures are properly cited

* All relevant supporting information is included in the manuscript submission,

* There are no issues that prevent the paper from being properly typeset

You will receive further instructions from the production team, including instructions on how to review your proof when it is ready. Please keep in mind that we are working through a large volume of accepted articles, so please give us a few days to review your paper and let you know the next and final steps.

Lastly, if your institution or institutions have a press office, please let them know about your upcoming paper now to help maximize its impact. If they'll be preparing press materials, please inform our press team within the next 48 hours. Your manuscript will remain under strict press embargo until 2 pm Eastern Time on the date of publication. For more information, please contact onepress@plos.org.

You will receive an invoice from PLOS for your publication fee after your manuscript has reached the completed accept phase. If you receive an email requesting payment before acceptance or for any other service, this may be a phishing scheme. Learn how to identify phishing emails and protect your accounts at https://explore.plos.org/phishing.

If we can help with anything else, please email us at customercare@plos.org.

Thank you for submitting your work to PLOS ONE and supporting open access.

Kind regards,

PLOS ONE Editorial Office Staff

on behalf of

Dr. Gaurav Arora

Academic Editor

PLOS One
